# Construction of PANoptosis signature: Novel target discovery for prostate cancer immunotherapy

**DOI:** 10.1016/j.omtn.2023.07.010

**Published:** 2023-07-15

**Authors:** Xianyanling Yi, Jin Li, Xiaonan Zheng, Hang Xu, Dazhou Liao, Tianyi Zhang, Qiang Wei, Hong Li, Jiajie Peng, Jianzhong Ai

**Affiliations:** 1 Department of Urology, Institute of Urology, West China Hospital, Sichuan University, 88 South Keyuan Road, Chengdu 610041, China; 2 School of Computer Science, Northwestern Polytechnical University, Xi’an 710072, China

**Keywords:** MT: Bioinformatics, PANoptosis, pyroptosis, apoptosis, necroptosis, prostate cancer, immunotherapy, construction, novel target

## Abstract

PANoptosis pathway gene sets encompassing pyroptosis, apoptosis, and necroptosis were identified from the MSigDB database. We analyzed the perturbations and crosstalk in the PANoptosis pathway in prostate adenocarcinoma (PRAD), including gene mutation, transcription, methylation, and clinical features. By constructing a PANoptosis signature, we accurately predicted the prognosis and immunotherapeutic response of PRAD patients. We further explored the molecular features and immunological roles of the signature, dividing patients into high- and low-score groups. Notably, the high-score group correlated with better survival outcomes and immunotherapeutic responses, as well as a higher mutation frequency and enrichment score in the PANoptosis and HALLMARK pathways. The PANoptosis signature also enhanced overall antitumor immunity, promoted immune cell infiltration, upregulated immune checkpoint regulators, and revealed the cold tumor characteristics of PRAD. We also identified potential drug targets based on the PANoptosis signature. These findings lead the way in identifying novel prognostic markers and therapeutic targets for patients with PRAD.

## Graphical abstract

**Figure:**
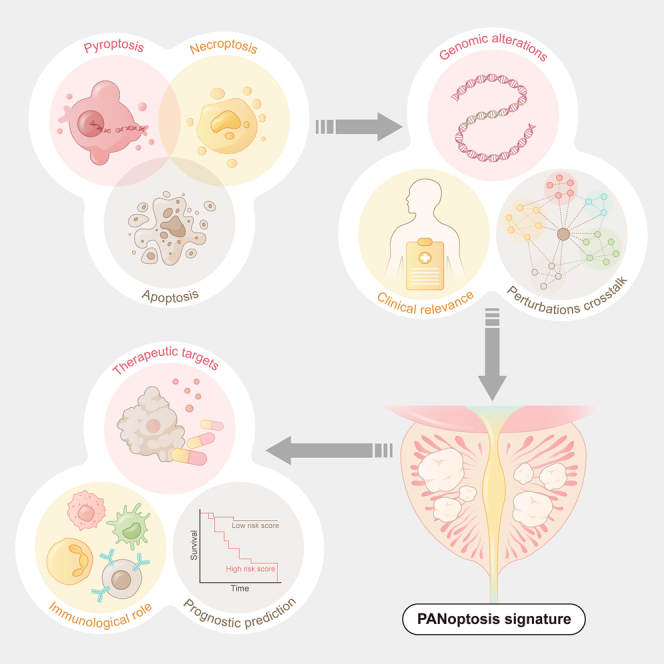

## Teaser

Jianzhong Ai and colleagues describe the profile of PANoptosis in PRAD. They construct a PANoptosis signature, which excellently predicts the prognosis and immunotherapeutic response, and reveal the cold tumor characteristics of PRAD. These findings pave an avenue for identification of novel therapeutic targets for PRAD.

## Introduction

Aging increases the risk of morbidity due to prostate adenocarcinoma (PRAD).^1^ In the United States, the number of PRAD cases is expected to reach 268,490 in 2022, and the number of deaths ranks second among patients with cancer.^2^ Radical prostatectomy, radiotherapeutic treatment, and androgen deprivation therapy are recommended for patients with low- or intermediate-risk PRAD.^3^^,^^4^ Furthermore, immunotherapy, including immune checkpoint inhibitor therapy and chimeric antigen receptor T cell therapy, could improve the antitumor effect and overall survival (OS) in patients with advanced PRAD.^5^^,^^6^ However, despite the considerable efficacy of the therapeutic methods used, PRAD manifests as a “cold tumor,” with decreased antigen expression, defective tumor suppression, and poor immunological infiltration.^7^ As a result, some subtypes of patients with PRAD are less responsive to immunotherapy, progress to drug resistance, cancer recurrence, and metastasis and are prone to a poor prognosis.^8^ Therefore, exploiting novel interventions and improving management to improve patient prognosis is urgent. Apart from developing innovative treatments, using effective biomarkers, targets, and signatures might help overcome the obstacles of PRAD. Among them, targeting cell death pathways is a feasible option. Cell death is vital for organismal homeostasis in humans. Pyroptosis, apoptosis, and necroptosis are important programmed cell death (PCD) pathways. Mounting evidence indicates extensive interactions between these pathways,^9^^,^^10^^,^^11^^,^^12^ and PANoptosis (named after pyroptosis, apoptosis, and necroptosis) exhibits key genetic and molecular characteristics of the three PCD pathways.^9^^,^^10^^,^^11^^,^^13^ Triggered by Z-DNA binding protein 1 (ZBP1) and mediated by the PANoptosome complex,^9^^,^^14^ PANoptosis is activated in various infectious diseases, such as influenza A virus infection. Although evidence for the effects of PANoptosis on PRAD is scarce, the three PCD pathways have been implicated in tumors. For instance, apoptosis maintains membrane integrity, preventing the leakage of cellular contents and subsequent tissue injury and inflammation.^15^ Some studies have reported that pyroptosis inhibits tumorigenesis and tumor progression and regulates the tumor microenvironment (TME).^16^^,^^17^ Others have shown that pyroptosis may create a microenvironment that drives tumor progression.^16^^,^^18^ Necroptosis plays an important role in the antitumor TME,^19^ but necroptosis of tumor cells may also promote tumor metastasis.^20^ Therefore, PANoptosis is correlated with tumor characteristics and the prognosis of patients with tumors. Studies have separately examined the predictive value of pyroptosis-, apoptosis-, and necroptosis-related models in specific tumor types with heterogeneous effectiveness,^21^^,^^22^^,^^23^ but studies on the effect of PANoptosis on patients with tumors are lacking.

In our study, the PANoptosis signature was delineated with bioinformatics analysis. Using this PANoptosis signature, we evaluated the potential correlation between PANoptosis and PRAD characteristics and its predictive value for therapeutic response and prognosis. Our findings could provide innovative targeted therapies for treating patients with PRAD.

## Results

### Perturbations and crosstalk in PANoptosis in PRAD

**Figure 1:**
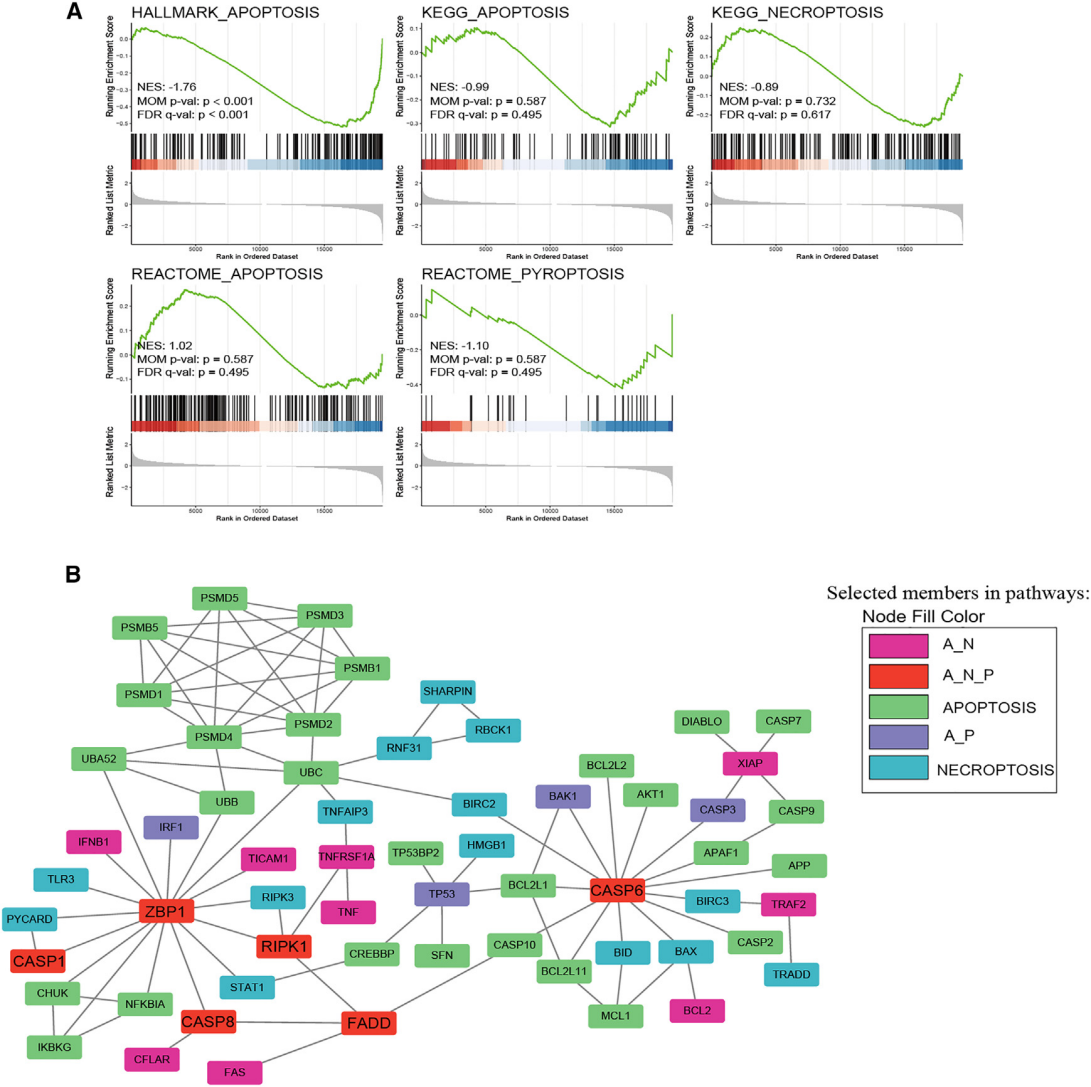
Perturbations and crosstalk in the PANoptosis pathway in PRAD (A) The GSEA enrichment plot shows differential enrichment between tumor and control groups in “REACTOME_PYROPTOSIS, HALLMARK_APOPTOSIS, KEGG_APOPTOSIS, REACTOME_APOPTOSIS, and KEGG_NECROPTOSIS.” (B) Network of proteins belonging to three death modalities. A_N, proteins involved in apoptosis and necroptosis; A_P, proteins involved in apoptosis and pyroptosis; A_N_P, proteins involved in apoptosis, pyroptosis, and necroptosis.

To examine the perturbations in apoptosis, pyroptosis, and necroptosis in tumors, we compared differentially enriched pathways between tumors and adjacent normal tissues using gene set enrichment analysis (GSEA) on five PANoptosis pathways (REACTOME_PYROPTOSIS, HALLMARK_APOPTOSIS, KEGG_ APOPTOSIS, REACTOME_APOPTOSIS, and KEGG_NECROPTOSIS) (Figure 1A). Similarly, we analyzed differences in enrichment in patients aged between ≥60 and <60 years. The results indicated that five PANoptosis pathways were more abundant in adjacent normal tissues or patients aged ≥60 years, although statistical significance was observed only in the HALLMARK_APOPTOSIS pathway (Figure S1A).

Next, we constructed an interaction network to examine the crosstalk between apoptosis, pyroptosis, and necroptosis using the STRING database. The network graph showed that each cell death pathway was unlikely to be independent of other pathways, supporting the concept of a unified death network (Figure 1B). Notably, apoptosis, pyroptosis, and necroptosis were interconnected by a set of genes, such as ZBP1, RIPK1, CASP6, CASP1, CASP8, and FADD. These results confirmed the existence of perturbations and crosstalk of PANoptosis in PRAD. Therefore, we evaluated the PANoptosis patterns in various aspects of PRAD.

### Status of gene mutation and methylation of PANoptosis pathway genes in PRAD

**Figure 2:**
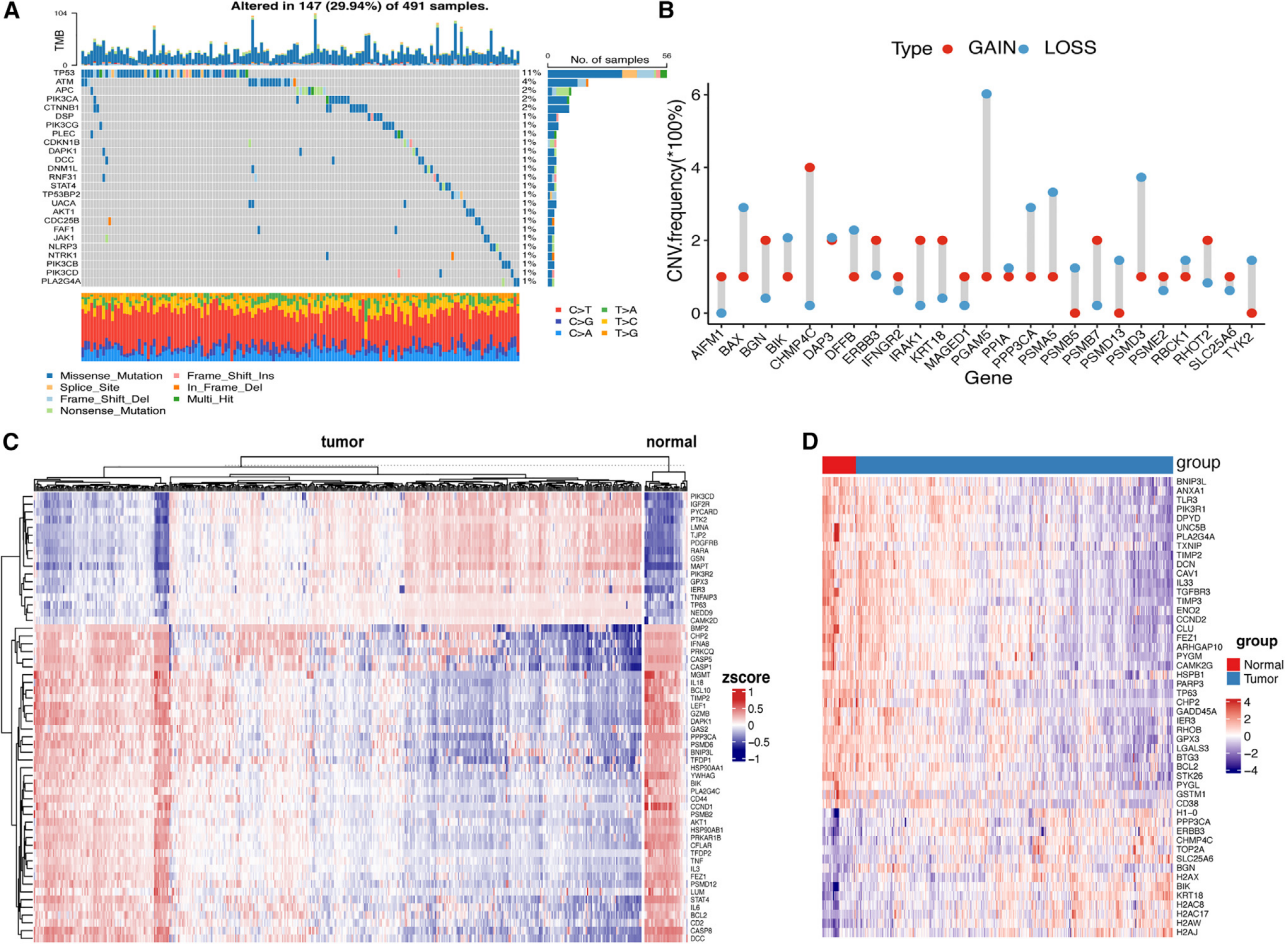
Genomic alterations in the PANoptosis pathway in PRAD (A) Mutational landscape of the top 25 mutated genes in PANoptosis. (B) Dumbbell diagram shows the top 25 PANoptosis pathway genes significantly upregulated in tumors. (C) Heatmap of methylation of PANoptosis pathway genes in tumors and controls. (D) Heatmap showing the top 50 differentially expressed genes in the PANoptosis pathway in tumor and control samples.

We visualized mutation data of TCGA-PRAD. The top 25 mutations of PANoptosis pathway genes are shown (Figure 2A). The most common mutations included cytosine mutations, which indicates that cytosine is unstable, possibly due to the facile oxidation of its amino groups. In addition, the overall mutation rate of PANoptosis pathway genes was not high, and the mutation frequency was <2% for all, except *TP53* and ATM, which had 11% and 4% mutation frequencies, respectively. A dumbbell diagram showed that the top 25 PANoptosis pathway genes were significantly upregulated in tumors (log2FC > 0, p < 0.05) (Figure 2B). Moreover, a Circos plot was used to visualize the distribution of the top 25 significantly upregulated genes on chromosomes (Figure S1B).

Next, we analyzed alterations in the methylation state of PANoptosis genes, and the heatmap graph (Figure 2C) showed differences in methylation levels of the top 30 PANoptosis pathway genes between tumors and controls. From the heatmap, we clearly observed that patients with tumors could be separated into two subgroups (Table S5). Kaplan-Meier (KM) survival curves revealed no significant differences in OS between the subgroups (Figure S2A). However, we found that the expression levels of most immune checkpoint regulators were higher in cluster 1 (Figure S2B).

### Clinical relevance analysis of the PANoptosis pathway

Gene expression profiling data and clinical information from patients with PRAD were downloaded from The Cancer Genome Atlas (TCGA) database. We compared PANoptosis pathway gene expression levels between the tumor and normal adjacent tissues and found the top 50 genes in the heatmap (Figure 2D). Then, we grouped the patients based on age (ages ≥60 and <60 years) and examined gene expression differences between these groups. Figure S3A shows the expression levels of PANoptosis pathway genes (SEPTIN4, PDGFRB, HMOX1, BGN, DNAJC3, and LMNB1). The top 6 genes had the smallest p value and were significantly differentially expressed between patients aged ≥60 and <60 years (other genes are shown in Figure S3B). In addition, the patients were divided into different groups based on clinical parameters, including race (Asia vs. White vs. Black), seminal vesicle invasion (yes vs. no), and *TP53* mutational status (mutation vs. wild type), to analyze differences in PANoptosis pathway gene expression. The results are shown in Figures S4A–S4C. Thus, we conclude that associations exist between the expression of PANoptosis pathway genes and variations in clinical features.

**Figure 3:**
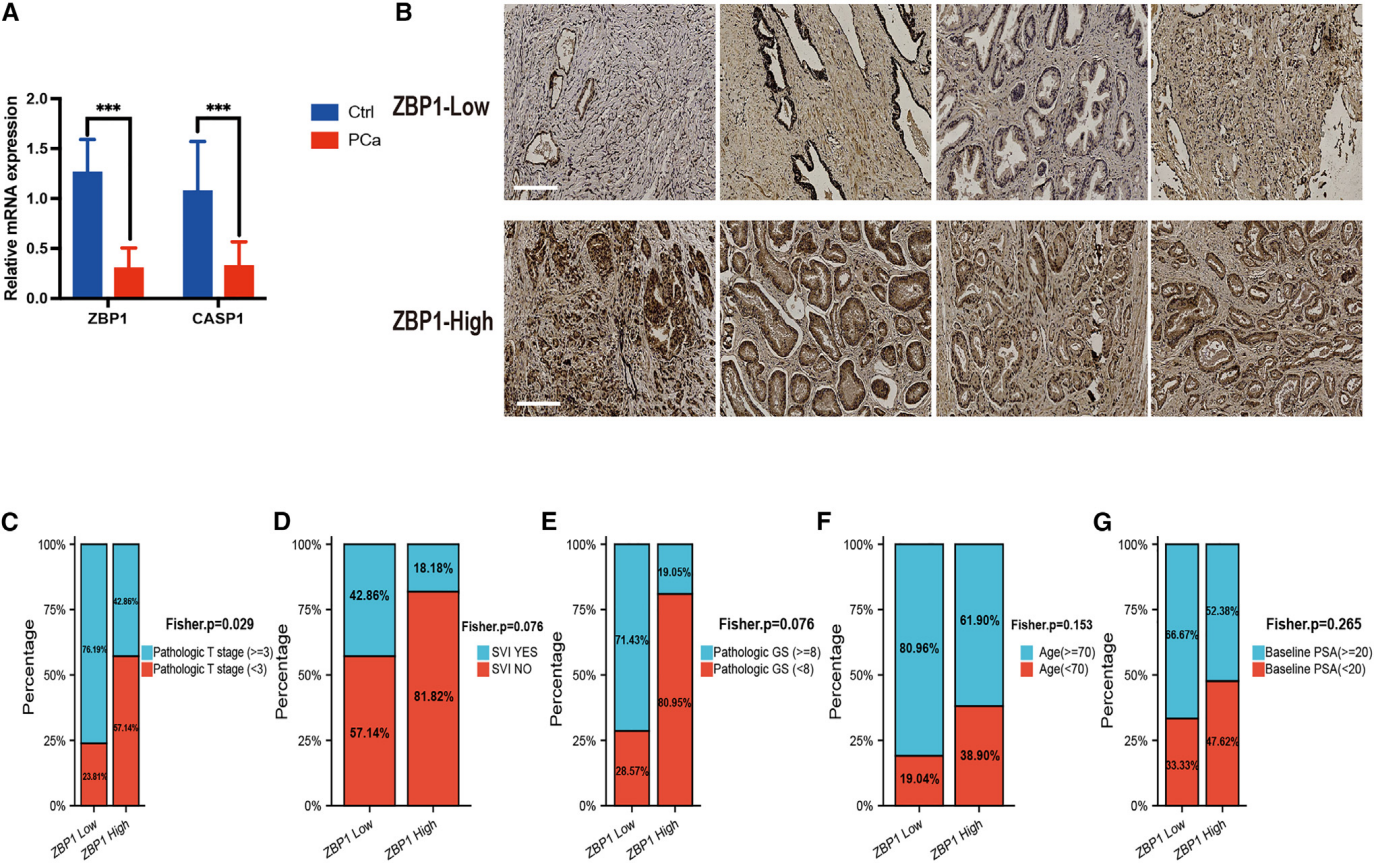
Analysis of the clinical relevance of PANoptosis (A) qPCR for PANoptosis key molecules. (B) Prostate cancer tissue specimens were analyzed by immunohistochemical staining using the anti-ZBP1 antibody. (C–G) Different clinical features with high and low expression of ZBP1. ZBP1, Z-DNA binding protein 1; SVI, seminal vesicle invasion; GS, Gleason score; PSA, prostate-specific antigen.

Further investigation was conducted using samples from PRAD and benign prostate hyperplasia (BPH) patients at West China Hospital of Sichuan University. PRAD and BPH samples were analyzed by nucleic acid extraction and PCR, and the expression levels of key molecules in PANoptosis, including ZBP1, RIPK1, CASP6, CASP1, CASP8, and FADD, were identified. The results showed that the expression of ZBP1 and CASP1 in PRAD tissues was significantly lower than that in the BPH group, while the expression levels of CASP6 and RIPK1 were not different between the two groups (Figures 3A and S3C). qRT-PCR results indicated that the cycle threshold values of CASP8 and FADD were higher than 30; therefore, further analysis was not conducted. Next, we used the anti-ZBP1 antibody for immunohistochemistry (IHC). Patients were divided into high- and low-ZBP1-expressing groups based on the positive staining score (Figure 3B). Different clinical features were observed in the two groups (Figures 3C–3G). High ZBP1 expression was significantly associated with lower pathologic T. A similar trend was found for other clinical features (seminal vesicle invasion, pathologic Gleason score, age, and baseline prostate-specific antigen [PSA]), although these differences did not reach statistical significance (p = 0.076, 0.076, 0.153, and 0.265, respectively). Overall, high expression of ZBP1 was more likely related to better clinical features.

Based on these results, we evaluated overlapping genes from the differentially methylated site, differentially expressed transcriptome, and mutated datasets. Nine overlapping genes were obtained by taking the intersection of 142 differentially methylated sites, 68 differentially expressed transcriptome genes, and the top 100 mutated genes. Methylation status, differential expression, and gene mutations are shown in Figures S5A–S5C, respectively.

### PANoptosis affects the prognosis of patients with PRAD

Patients were separated into high- and low-expression groups according to the median expression levels of PANoptosis pathway genes. Using survival data from the TCGA database, we compared OS between groups. Eight PANoptosis pathway genes showed differences in OS between high- and low-expression groups, and the KM survival curves were ranked based on their p values (Figure S6). Specifically, patients with high expression of TP53, HSP90AA1, PSMD5, PSMD3, and GAS2 had significantly worse OS than those with low expression, whereas patients with high expression of APP, CASP4, and VDAC3 had a more favorable OS.

Although there was only a statistical significance in HALLMARK_APOPTOSIS by GSEA analysis, the crosstalk between the three modes of death was found, which indicated they were unlikely to be independent of the others. Moreover, characterization of the PANoptosis pathway including mutation, transcription, methylation, and clinical relevance presents difference in PRAD and controls, especially its expression pattern is closely related to prognosis, which is of great significance for studies on PANoptosis. Therefore, we constructed a PANoptosis signature using data from 52 patients who developed biochemical recurrence (BCR) (from the TCGA database). The PANoptosis signature score was obtained by calculating the mean enrichment scores of pyroptosis, apoptosis, and necroptosis. After that, 52 BCR patients were divided into a high signature score group and a low signature score group based on the median score.

### PANoptosis signature shows predictive value for PRAD prognosis

**Figure 4:**
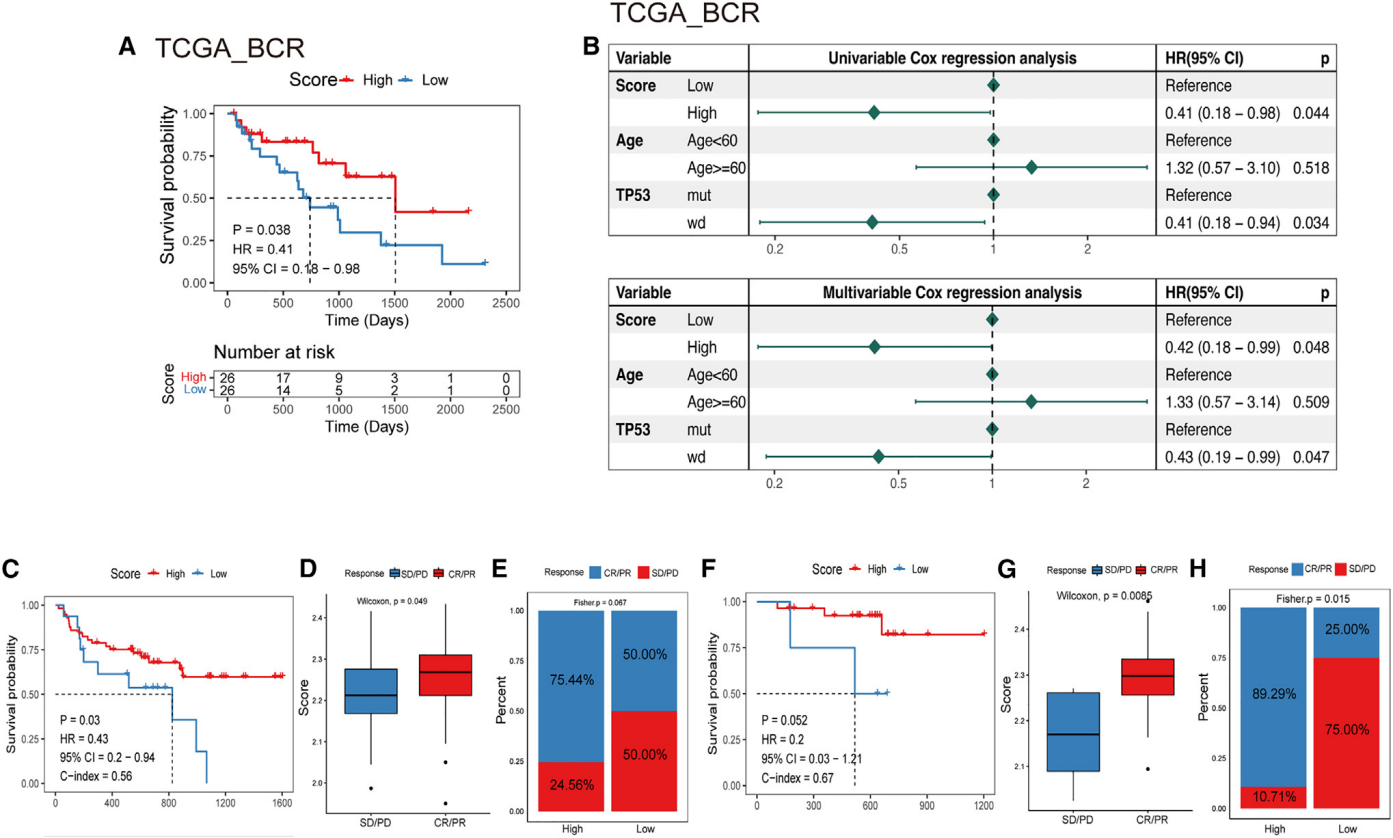
PANoptosis signature predicts the prognosis and immunotherapy response of patients with PRAD (A) The differences in survival probability between the high- and low-signature score groups. (B) Prognostic prediction of PANoptosis signature scores in the TCGA cohort. (C) Survival outcomes in melanoma patients treated with anti-PD-1 monotherapy (PMID30753825). (D and E) Correlation between the PANoptosis signature score and clinical response to cancer immunotherapy in patients with melanoma treated with anti-PD-1 monotherapy (PMID30753825). (F) Survival outcomes in melanoma patients treated with combined anti-PD-1 and anti-CTLA-4 therapy. (G and H) Correlation between the PANoptosis signature score and clinical response to cancer immunotherapy in patients treated with combined anti-PD-1 and anti-CTLA-4 therapy. CR, complete response; PR, partial response; PD, progressed disease; SD, stable disease. CR/PR, patient with CR or PR; SD/PD, patient with SD or PD.

We took advantage of survival data collected by TCGA and plotted KM survival curves for the training cohort. The results revealed that patients with low signature scores had significantly lower odds of survival than patients with high signature scores, suggesting that patients with high scores have better clinical outcomes in the training cohort (Figure 4A).

The risk model was then validated using another three sets of validation datasets. The validation cohort GSE21034 was grouped by median score, and a significant difference was observed in OS between the high- and low-score groups (Figure S7A). In addition, the same trend was found in the validation cohorts PRAD-FR_seq_RFS and GSE54460 when patients were grouped by the optimal grouping method (Figures S7B and S7C).

Next, we performed univariable and multivariable Cox regression analyses to determine whether the signature score was an independent prognostic factor affecting survival. Univariate Cox proportional hazard regression analysis showed that the signature score had a hazard ratio < 1 in the TCGA cohort and was significantly associated with BCR in the TCGA cohort. Moreover, the signature score remained an independent protective predictor of BCR risk after adjusting for other prognostic factors (Figure 4B). In the validation cohort, the results were consistent in the univariate analysis and multivariable analysis for no information on other clinical features (Figures S7D–S7F). From these results, we propose that the PANoptosis signature can predict the prognosis of PRAD. Moreover, the PANoptosis signature score is a protective factor and is positively related to the survival outcome of patients.

In addition, we compared clinical features between the high- and low-score groups by using clinical information from the TCGA database and cohort GSE21034 and found no significant differences in clinical features between the two groups (Figure S8).

### PANoptosis signature predicts immunotherapeutic response

To determine the predictive ability of the tumor PANoptosis signature score in predicting benefit from immunotherapy, we collected immunotherapeutic response data from a melanoma cohort (PMID30753825). Patients with melanoma in this cohort were treated with anti-PD-1 monotherapy (n = 63) or combined anti-PD-1 and anti-CTLA-4 therapy (n = 57). After calculating the PANoptosis signature score, patients were divided into high- and low-score groups. KM curve analysis revealed that patients treated with anti-PD-1 monotherapy who had high signature scores had better survival outcomes (Figure 4C). A similar result was found in patients treated with combined anti-PD-1 and anti-CTLA-4 therapy (Figure 4F), but the differences were not significant (p = 0.052). Subsequently, we evaluated the PANoptosis signature score in the partial response (PR)/complete response (CR) and stable disease (SD)/progressive disease (PD) groups. The PANoptosis signature was shown to have a higher score in the PR/CR group than in the SD/PD group (Figures 4D and 4G). Similarly, when patients treated with combined anti-PD-1 and anti-CTLA-4 therapy were divided into high- and low-score groups, PR or CR in the high-score group accounted for 89.29%, and PR or CR in the low-score group accounted for only 25% (Figure 4H). The same trend was observed in patients treated with anti-PD-1 monotherapy (Figure 4E). Our PANoptosis signature has good predictive value for the immunotherapeutic response.

### Characteristics of the PANoptosis model regarding genomic alterations and enrichment pathways

**Figure 5:**
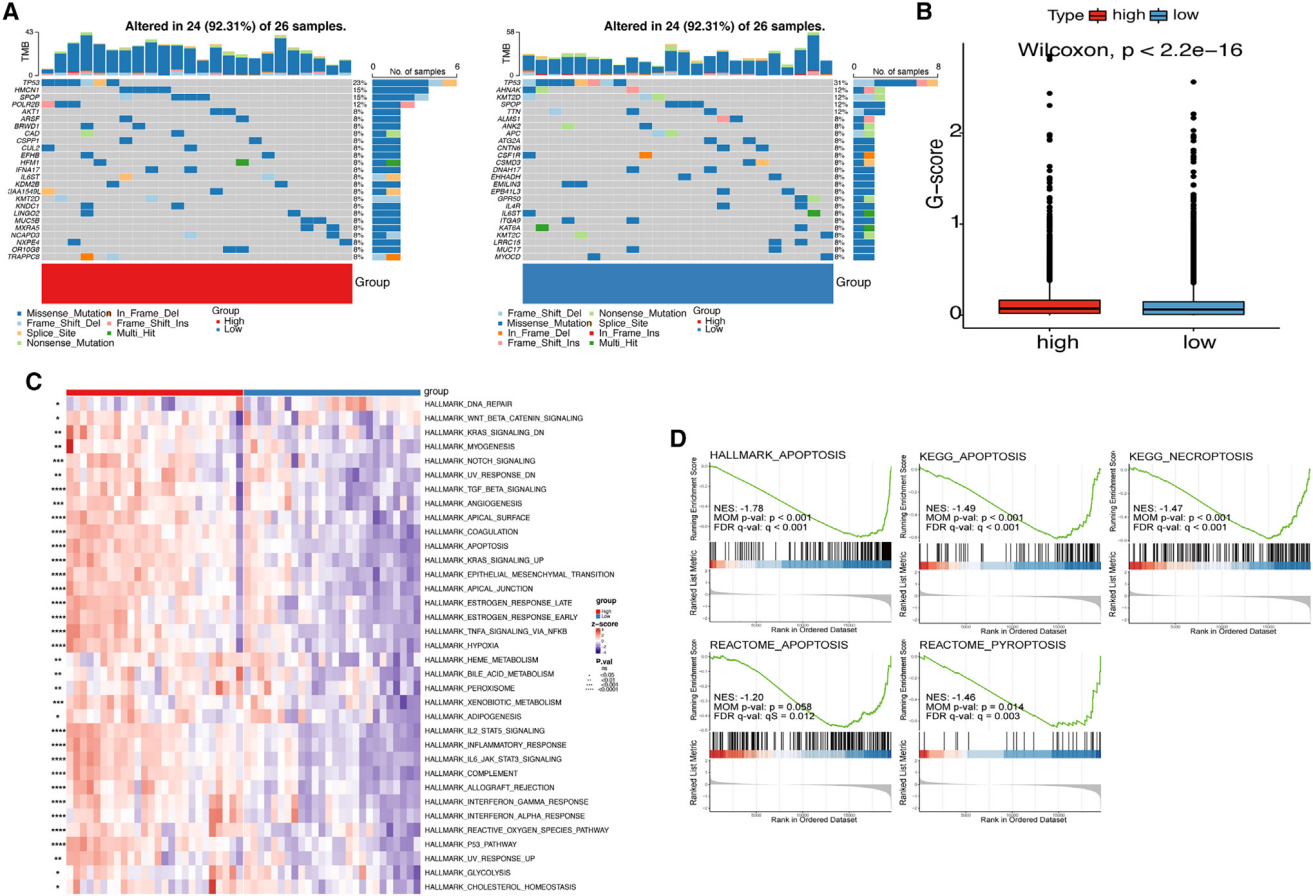
Genomic alterations and pathway enrichment in the PANoptosis signature (A) The mutational landscape of PANoptosis pathway genes altered between high- and low-risk patients with PRAD in the TCGA cohort. (B) The frequency difference in copy-number amplification and copy-number deletion. (C) The enrichment score of hallmark pathways in the high- and low-risk score groups. (D) GSEA of enrichment differences in the three death modes between the high- and low-risk score groups. G scores, The Genomic Identification of Significant Targets in Cancer score; ∗p ≤ 0.05, ∗∗p ≤ 0.01, ∗∗∗p ≤ 0.001, ∗∗∗∗p ≤ 0.0001.

We used single-nucleotide variant (SNV) and copy-number variation (CNV) data from TCGA database to evaluate genomic alterations between different signature score groups. Mutations in all PANoptosis pathway genes in TCGA-PRAD are shown in Figures 5A and S9A. In patients with BCR, the high- and low-score groups had a 92.31% rate of overall PANoptosis gene mutation, but the mutation rate of *TP53* was higher in the low-score group. In all TCGA patients with PRAD, the mutation rate was only 8.15%, which is much lower than that in patients with BCR. More results of mutation differences between the high- and low-score groups are shown in Table S6. The Genomic Identification of Significant Targets in Cancer (GISTIC) score (G scores) of genomic segments of high- and low-signature score groups plotted along chromosomes are shown in Figures S9B and S9C, respectively. Amplifications are in red and deletions are in blue. The frequency difference in copy-number amplification and copy-number deletion between groups is shown in Figure 5B. We found that the mutation frequency was significantly higher in the high-signature score group.

Single-sample GSEA (ssGSEA) was used to calculate HALLMARK enrichment scores. Then, the HALLMARK pathway enrichment score was compared between the high- and low-score groups. A significant difference was observed between the groups in almost all HALLMARK pathways, and the HALLMARK pathways were more enriched in the high-signature score group (Figure 5C).

GSEA was used to analyze enrichment differences in three death modes between groups. HALLMARK_APOPTOSIS, KEGG_NECROPTOSIS, KEGG_APOPTOSIS, REACTOME_PYROPTOSIS, and REACTOME_APOPTOSIS showed significant enrichment of differences between the high- and low-signature score groups. These results indicated that apoptosis, pyroptosis, and necroptosis were significantly inhibited in the low-PANoptosis signature score group compared with the high-PANoptosis signature score group (Figure 5D).

Then, we evaluated the ZBP1-mediated PANoptosis pathway. The expression levels of 11 genes from the classic ZBP1-mediated PANoptosis pathway were compared between the high- and low-score groups. Our results revealed that the expression level of most genes was significantly higher in the high signature score group (Figure S10A). Subsequently, Spearman’s analysis indicated that 10 genes from the classic ZBP1-mediated PANoptosis pathway were significantly positively related to the PANoptosis signature score (Figure S10B). These results indicated that the PANoptosis signature was tightly related to the ZBP1-mediated PANoptosis pathway.

### PANoptosis boosts tumor-specific immunity in PRAD

**Figure 6:**
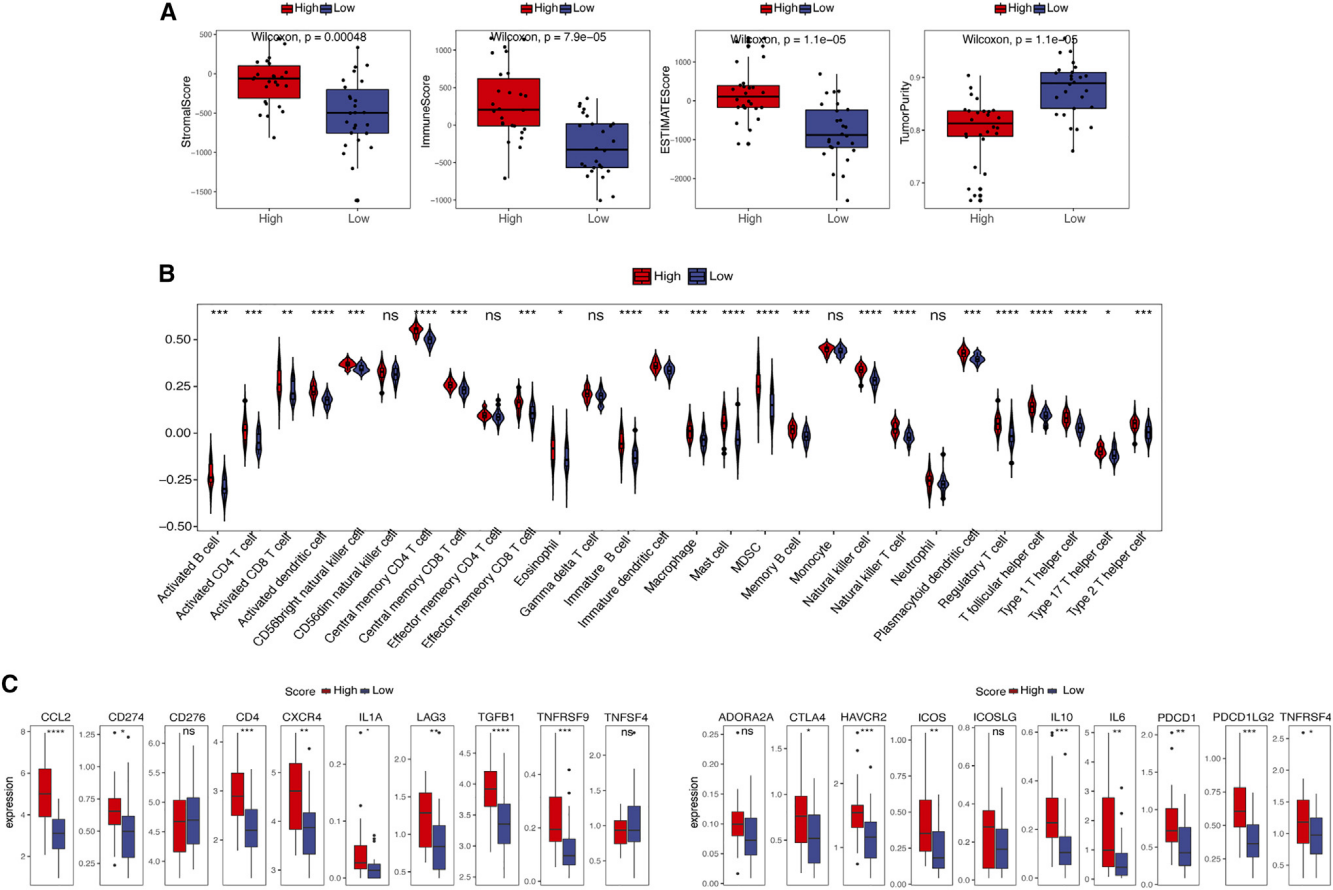
Immunological role of the PANoptosis signature in PRAD (A) An ImmuneScore, StromalScore, and EstimateScore in the high- and low-score groups. (B) Enrichment scores of 28 immune cell infiltrates in the high- and low-score groups. (C) Expression levels of immune checkpoint regulators in the high- and low-score groups in the TCGA cohort. ∗p ≤ 0.05, ∗∗p ≤ 0.01, ∗∗∗p ≤ 0.001, ∗∗∗∗p ≤ 0.0001; ns, not significant.

ImmuneScore, StromalScore, EstimateScore, and TumorPurity were calculated with ESTIMATE. The boxplots showed that the ImmuneScore, StromalScore, and EstimateScore were significantly higher in the high-score group, whereas the TumorPurity score was significantly lower (Figure 6A). To further understand and characterize the immune microenvironment in relation to the PANoptosis signature score, the profile of TME cell infiltration models was evaluated. ssGSEA was used to quantify the relative abundance of 28 immune cell infiltrates. We found significant differences in immune cell infiltration in the subgroups (Figure 6B). A high PANoptosis signature score correlated with infiltration of immune cells, such as CD4, CD8, and natural killer cells, in the TME, which promote tumor immunity. However, the infiltration of regulatory T (Treg) cells, which suppress tumor immunity,^24^ was also positively correlated with the PANoptosis signature score.

Immune checkpoints regulate the level of immune activation. Our results revealed that the expression levels of some immune checkpoints, such as CCL2, CD274, CD4, CXCR4, and LAG3, were significantly higher in the high-score group than in the low-score group (Figure 6C). To better understand the role of the PANoptosis signature in tumor immune escape, we evaluated tumor immunogenicity, including tumor mutation burden (TMB), neoantigen load, homologous recombination deficiency (HRD), loss of heterozygosity (LOH), and cancer/testis antigens (CTAs). Significant differences were seen between the two score groups only at the CTA level (Figure S11), indicating that the tumor cells could recognize more antigens and thus initiate an immune response in the high-score group. Overall, the PANoptosis signature plays significant roles in PRAD tumor immunity because it promotes immune cell infiltration, increases the expression of immune checkpoint regulators, and promotes tumor immunogenicity.

### PANoptosis provides potential therapeutic targets for the effective treatment of PRAD

**Figure 7:**
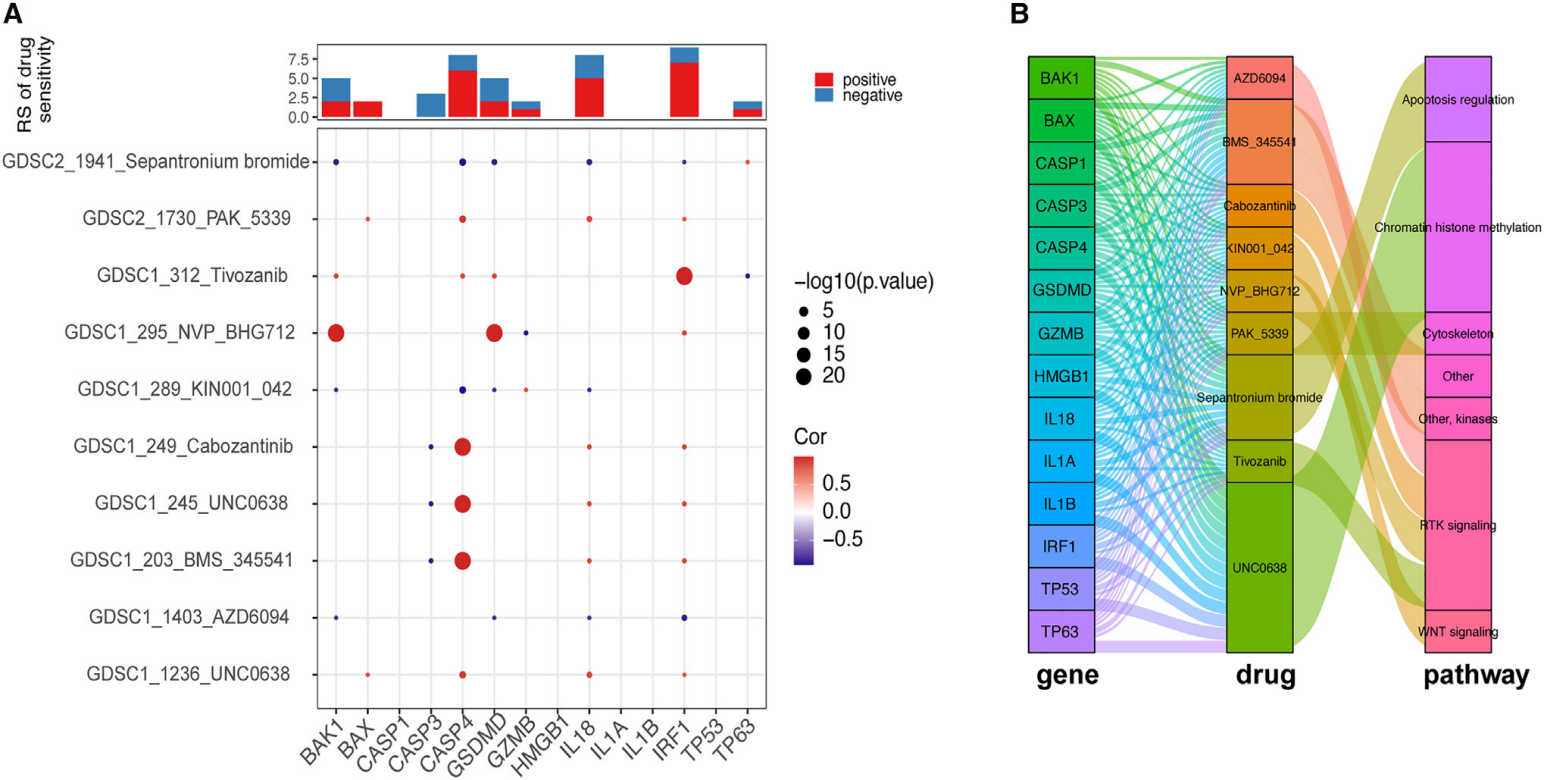
Predictive value of PANoptosis signature-based therapeutics in PRAD (A) Correlations between the PANoptosis signature score and drug sensitivity. The value of the correlation coefficient is represented by the intensity of blue or red, as indicated on the color scale. (B) The Sankey diagram demonstrates the relationship between PANoptosis genes, drugs, and drug-related pathways.

We evaluated differentially expressed genes between signature score groups and performed pathway enrichment analysis. Then, by using the drug response data obtained from the Genomics of Drug Sensitivity in Cancer database, we acquired the half-maximal inhibitory concentration (IC_50_) values of some drugs used in PRAD based on PANoptosis signature gene expression (Figure S12). Spearman’s correlations between the expression of PANoptosis signature genes and drug sensitivity are shown in Figure 7A. Notably, CASP4 strongly positively correlated with sensitivity to several drugs, such as BMS_345541, UNC0638, and cabozantinib. BAK1 and GSDMD were strongly positively correlated with sensitivity to BHG712, and IRF1 was strongly positively correlated with sensitivity to tivozanib. Sankey diagrams were used to visualize the relationship between PANoptosis genes, drugs, and drug-related pathways (Figure 7B). For PANoptosis genes, we targeted nine drugs and seven pathways involved in drug action. Our results demonstrated that sepantronium bromide plays a role in tumor suppression through apoptosis regulation by targeting a series of PANoptosis genes. In addition, these PANoptosis genes act as drug-targeted genes through various signaling pathways.

### Profile analysis of the PANoptosis signature in prostate cells

**Figure 8:**
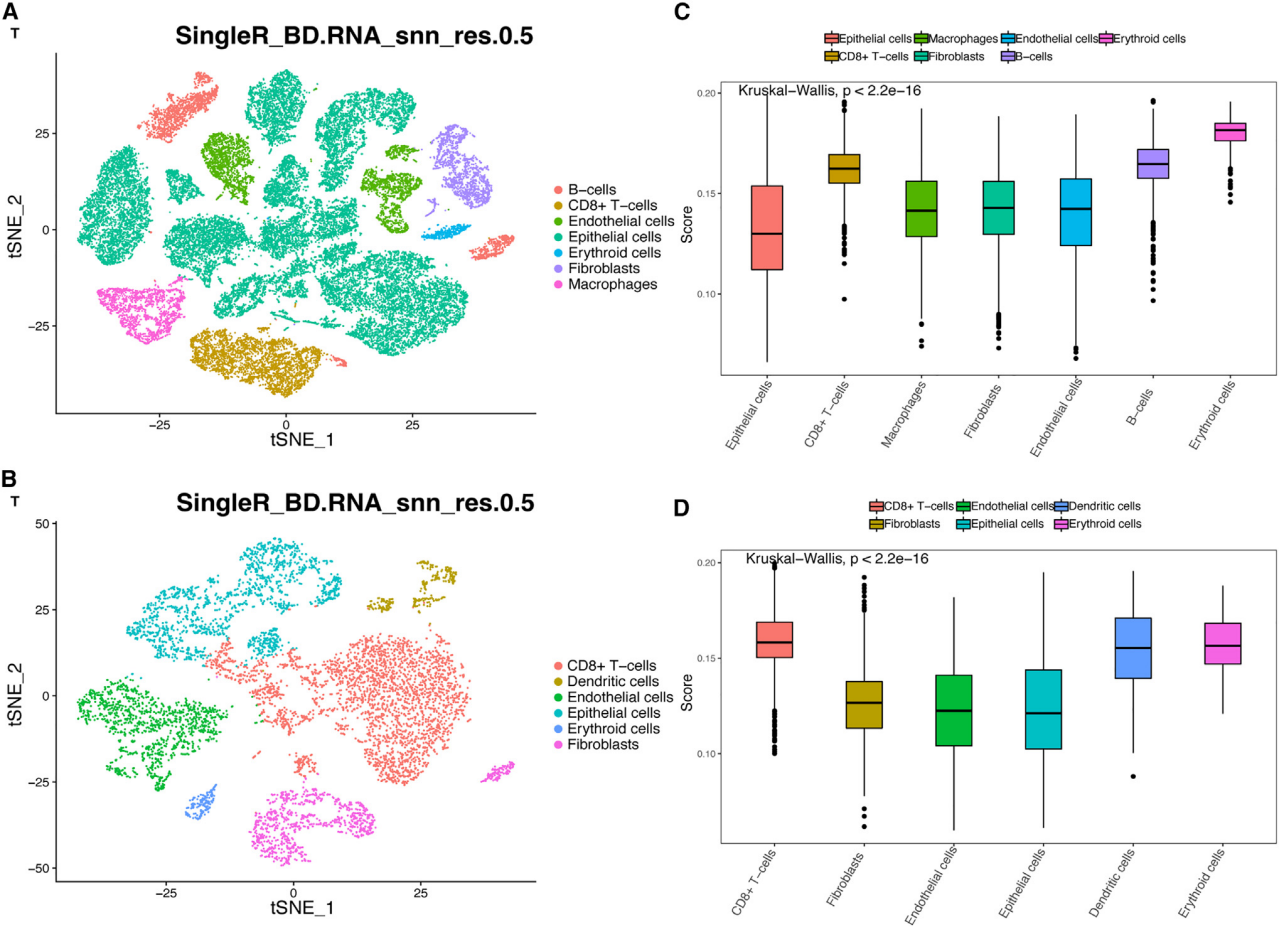
Profile of the PANoptosis signature in PRAD using single-cell transcriptomic analysis (A and B) A t-distributed stochastic neighbor embedding (t-SNE) view of two scRNA-seq profiles (GSE141445 and GSE157703). (C and D) PANoptosis signature score in various cell types using data from the GSE141445 and GSE157703 cohorts.

To further evaluate the profile of the PANoptosis signature in PRAD, we performed single-cell transcriptome analysis using data obtained from the Gene Expression Omnibus (GEO) database. Based on PRAD samples from single-cell datasets (GSE141445 and GSE157703), t-distributed stochastic neighbor embedding (t-SNE) clustering of cells, and cell group annotation were performed (Figures 8A and 8B). Moreover, we calculated the PANoptosis signature score in various cell types and found that the PANoptosis signature score varied across cell types (Figures 8C and 8D). Notably, in the two cohorts, the PANoptosis signature score was high in CD8+ T cells, B cells, and dendritic cells, indicating that these cells had more severe PANoptosis.

## Discussion

Up to 20%–40% of patients with localized PRAD develop recurrent disease within 10 years,^25^ and nearly all patients with advanced PRAD develop castration-resistant prostate cancer, despite most having initially robust responses to androgen deprivation therapy.^26^^,^^27^ Many efforts have been made to improve the efficacy of PRAD treatment, such as immunotherapy, which has emerged as a viable treatment option. However, PRAD is not highly responsive to immunotherapy because of its molecular characteristics and the key role that various signaling pathways play in the development of drug resistance and immunosuppression.^5^^,^^28^ Therefore, the prognosis of advanced castration-resistant prostate cancer remains lethal, and there is an urgent need to develop biomarkers that predict patient prognosis and provide new therapeutic targets for treating PRAD. Recently, the concept of PANoptosis was introduced. PANoptosis is mediated by the PANoptosome complex and features all the characteristics of pyroptosis, necroptosis, and apoptosis.^9^ PANoptosis plays a crucial role in the development of cancer, particularly in tumor immunity.^29^^,^^30^^,^^31^ Beretta and Zaffaroni summarized the molecular mechanisms involved in necroptosis and discussed the therapeutic potential for prostate cancer.^32^ Westaby et al. discussed the latest approaches for targeting the intrinsic apoptosis pathway for treating prostate cancer.^33^ However, these studies did not integrate the three modes of death in prostate cancer. Our study is the first to evaluate the features of PANoptosis in PRAD. In this study, we illustrated the role of PANoptosis in the development and progression of PRAD. The PANoptosis signature was constructed to identify its prognostic value and develop new therapeutic strategies for PRAD.

The deregulation of apoptosis plays a crucial role in tumorigenesis. Our results indicate that the HALLMARK_APOPTOSIS pathway was significantly activated in adjacent normal tissues compared with tumor tissues. Although there were no significant differences between other death pathways, the same trends were observed. Our interaction network showed that each cell death pathway was unlikely to be independent of other pathways, supporting the concept of a unified death network. Previous studies have demonstrated the potential mechanisms of interactions.^11^^,^^12^^,^^34^ For instance, apoptosis and necroptosis are closely linked through caspase-8 activity, which is a typical activator of extrinsic apoptosis, but it also inhibits necroptosis signaling through the cleavage of RIPK1 and RIPK3.^35^^,^^36^ Both pyroptosis and necroptosis are inflammatory processes. MLKL, the terminal effector of necroptosis, can disrupt the membrane and activate the NLRP3 inflammasome, which induces pyroptosis.^37^ Both apoptosis and pyroptosis involve the activation of members of the caspase family of proteases, and they may be linked by caspase-1, caspase-8, etc. Therefore, the introduction of the concept of PANoptosis is meaningful because, when any program is blocked, others can complete cell death.

Mutation analysis of PANoptosis pathway genes in PRAD indicated that the overall mutation rate of PANoptosis pathway genes in PRAD was not high, which was consistent with the characteristics of prostate cancer as a cold tumor, with a relatively low mutation rate. PANoptosis pathway genes showed different expression models and prognostic impacts. Interestingly, most PANoptosis genes appear to be associated with better clinical features and survival outcomes; however, we could not provide a unifying explanation for all the genes. Therefore, further experiments were performed to integrate PANoptosis pathway genes and assess them more accurately. Particularly, considering the importance of some molecules, we used PRAD tissue and BPH tissue from West China Hospital to identify expression patterns, and anti-ZBP1 IHC was performed using prostate cancer tumor tissue samples. We experimentally validated that the expression levels of CASP-1 and ZBP1 were lower in PRAD tissues, and that the expression of ZBP1 positively correlated with better clinical features. There is evidence to suggest that CASP1 participates in cell survival signaling by serving as a scaffold to impact complex I assembly.^38^^,^^39^ ZBP1 is a member of a large multiprotein complex along with CASP1, CASP8, RIPK1, and other molecules, which drives PANoptosis.^40^ Through bioinformatics analysis and experimental validation, it was determined that ZBP1 and CASP1 function together in PANoptosis processes in PRAD. In addition, methylation data revealed that patients with tumors could be separated into two subgroups. Subsequently, we found that the expression levels of most immune checkpoint regulators were higher in cluster 1, although the mechanism involved is unclear.

We constructed a PANoptosis signature in this study. We found that patients with higher PANoptosis signature scores had better survival outcomes. We confirmed that the PANoptosis signature was an independent predictor for the prognosis of PRAD. From a clinical standpoint, the PANoptosis signature can be helpful for a more accurate prognosis, identifying patients with a poor prognosis and providing proactive interventions.

PANoptosis may stimulate the TME to achieve antitumor effects.^29^^,^^41^ Specifically, apoptosis is commonly weakened in the TME^42^ and, although it is not immunogenic, it can activate antitumor immunity under certain conditions, such as caspase deficiency.^43^^,^^44^ Necroptosis can promote dendritic cell maturation and enhance the antitumor ability of CD8 T cells.^45^^,^^46^ In addition, pyroptosis, as an immunogenic form of cell death, plays an antitumor role by producing proinflammatory cytokines to facilitate the infiltration of immune cells in the TME.^47^ In our study, the high PANoptosis signature group acquired significantly higher ImmuneScore, StromalScore, and EstimateScore and lower tumor purity, indicating that the high-score group had higher overall immunity. This result was consistent with the enrichment scores of immune cell infiltrates. The PANoptosis signature increased immune infiltration in the tumor, increasing antitumor immunity. In addition to the infiltration of effector T cells, activated T cells, and other antitumor immune cells, the infiltration of Treg cells also increased. Treg cells can inhibit the proliferation of effector T cells, secrete cytokines, and participate in the immune escape of some tumors. PANoptosis likely presents the characteristics of immunogenic cell death and produces proinflammatory cytokines, such as IL-1B and IL18, to promote the infiltration of immune cells into the TME. During this process, Treg cells, regulated by IL-10 and TGF, may increase infiltration. Moreover, a high PANoptosis signature score increased the expression of immune checkpoint regulators. Thus, PANoptosis promotes tumor immunity in PRAD. Importantly, a high PANoptosis signature score can improve the immune response to immunotherapy, and the PANoptosis signature score can predict the response of patients to immunotherapy. Combining all findings, improving the PANoptosis signature score seems to enhance antitumor immunity, improve the response rate of patients to immunotherapy, and improve the prognosis of patients. Thus, the PANoptosis signature score may provide a new direction for the treatment of prostate cancer.

An increasing number of antitumor drugs has been used to target the PANoptosis pathway with good efficacy.^33^^,^^48^^,^^49^^,^^50^ In this study, we evaluated the association between the PANoptosis signature and drug sensitivity and identified PANoptosis-related pathways and drugs that target these pathways. We identified potential therapeutic targets for prostate cancer based on the PANoptosis signature.

Finally, single-cell transcriptome analysis suggested that the PANoptosis signature score was high in CD8+ T cells, B cells, and dendritic cells. Theoretically, the activation of the PANoptosis signature will achieve more immune cell infiltration and a better immunotherapeutic effect. However, when the immune cell itself has a relatively high PANoptosis signature score, the immune efficacy will decrease. This finding may provide new insights into why PRAD acts as a cold tumor and does not respond well to immunotherapy. Therefore, strategies to effectively inhibit PANoptosis in those immune cells can enhance the immunotherapy of PRAD.

In conclusion, we evaluated the genomic, transcriptomic, and clinical features of the PANoptosis signature in PRAD. By constructing the PANoptosis signature, the prognosis and immunotherapeutic response of patients can be predicted. Our findings provide novel targets in the PANoptosis pathway for synergistically enhancing immunotherapeutic efficacy.

## Materials and Methods

### Data source and preprocessing

Clinical data and values of fragments per kilobase of transcript per million mapped reads from patients with PRAD were obtained from the TCGA database : http://cancergenome.nih.gov/^51^ using the TCGAbiollinks package in R. Gene IDs were converted using an ID conversion file (gencode.v38.annotation.gtf). If more than one Ensembl_ID matched the same Symbol_ID, the median value was selected for subsequent analyses. We obtained RNA-seq data of 481 primary tumor tissue samples and 51 adjacent normal tissue samples from the TCGA-PRAD dataset, and finally a total of 481 samples with both gene expression data and survival information was used for analysis. Of these patients, 52 developed BCR and were used for PANoptosis signature building. Tumor expression profiles and clinical information are shown in Table S1.

We collected five pathways downloaded from the MSigDB (V7.4) (GSEA | MSigDB: gsea-msigdb.org)^52^ database for analysis, including REACTOME_PYROPTOSIS, HALLMARK_APOPTOSIS, KEGG_APOPTOSIS, REACTOME_APOPTOSIS, and map04217 pathway (KEGG_NECROPTOSIS). The union of all gene sets for the five pathways was identified as the PANoptosis pathway gene set (Table S2). GSE21034, GSE54460, GSE141445, and GSE157703 were downloaded from the GEO database: https://www.ncbi.nlm.nih.gov/geo/,^53^ and the PRAD-FR-seq_RFS dataset was downloaded from the ICGC Data Portal: https://dcc.icgc.org/.^54^ In addition, an immunotherapy cohort of melanoma (PMID30753825) was obtained from Gide et al.’s study.^55^ Samples lacking survival information were excluded during data processing and analysis. The no-load probe and probes with more than one gene were removed and, if multiple probes corresponded to one gene, average expression levels of those probes were obtained.

### GSEA

PANoptosis genes included genes involved in apoptosis, pyroptosis, and necroptosis. GSEA is a computational method that determines whether *a priori* defined sets of genes show statistically significant, concordant differences between two biological states. GSEA was used to analyze differences in PANoptosis-related signaling pathway gene enrichment in tumors and adjacent normal tissue samples. Next, we evaluated enrichment differences in PANoptosis-related pathways between groups with clinical features, such as age (age ≥60 years vs. age <60 years). The gseaplot2 package in R was used to visualize significantly enriched PANoptosis pathway genes.

### PANoptosis-related gene interaction networks

Based on the genes involved in the PANoptosis pathway, the STRING database was used to construct the interaction network to evaluate the crosstalk of three death modes, namely, cell pyroptosis, cell apoptosis, and cell necrotizing apoptosis, in tumors.

### PANoptosis pathway mutation analysis and methylation analysis

For genes involved in the PANoptosis pathway, we assessed the frequency of mutation and the location of genes with CNV on the chromosome. We generated a waterfall plot dumbbell chart to show the status of gene mutation and CNV and constructed a Circos plot to represent the chromosomal distributions of genes. SNV data and CNV data were obtained from the TCGA database using TCGAbiollinks (Table S3). Prostate cancer mutation data in the TCGA database are in the form of a Mut2ation Annotation Format file. We used the maftools package in R for annotation analysis and visualization.

The pancancer methylation panel targets were selected for hypermethylated CpG sites in tumor vs. normal tissue based on TCGA data. TCGA-PRAD methylation data were downloaded from the TCGA Data Portal, and the methylation levels of genes involved in PANoptosis were analyzed between the tumor and normal groups. We further divided patients into two subgroups according to the methylation results. Then, we compared the survival outcomes between subgroups and evaluated the associations between immune checkpoints and subgroups.

### Association between PANoptosis pathway genes and clinical feature groups and tumor prognosis

The patients were grouped based on different clinical features, including race (Asian vs. White vs. Black), seminal vesicle invasion (yes vs. no), *TP53* mutational status (mutation vs. wild type), and age (age ≥60 years vs. age <60 years). We evaluated the differential expression of PANoptosis pathway genes between different groups. Similarly, we compared gene expression levels between tumor and normal adjacent tissues.

The patients were divided into high- and low-score subgroups based on the median PANoptosis pathway gene expression value, and then survival analysis was performed on the OS of subgroups using gene expression data and survival data from the TCGA database. The KM method and log rank test were used to evaluate differences in survival between patients with low or high levels of expression of PANoptosis pathway genes.

### Integrated analysis of mutation, transcription, and methylation of PANoptosis pathway genes

We set the threshold for |log2FC| > 0.585 and adj. A p value < 0.05 was used to identify differential gene expression at the transcript level, and 68 genes were included. A total of 142 differentially methylated sites and the top 100 mutant genes were obtained according to previously described methods. Then, 9 overlapping genes were obtained by accounting for their intersection in the analysis.

### Development of the PANoptosis signature for PRAD

ssGSEA was used to calculate the enrichment score of each sample. Mean enrichment scores for pyroptosis, apoptosis, and necroptosis were identified as PANoptosis signature scores.^56^^,^^57^^,^^58^^,^^59^ The risk score for each patient was calculated using the following formula.

$$Score=mean\;\left(NES_{P}+\sum_{i=1}^{n}NES_{A}i+NES_{N}\right)$$

where NES is the ssGSEA enrichment score, P is pyroptosis, A is apoptosis, N is necroptosis, i is apoptosis, and n is the number of apoptosis pathways.

For model construction, we used gene expression and survival data from 52 patients with BCR. The PANoptosis signature score of each patient was calculated based on the formula, and then patients were divided into high- and low-score groups according to the median score. The survminer package in R was used for survival analysis of BCR in the high- and low-score groups. The KM method was used to generate the survival curve, and the log rank test was used to compare differences between groups. Univariate and multivariate analyses were performed using Cox’s regression model, and the independent prognostic value of the PANoptosis signature score was determined in combination with other clinical features. p < 0.05 was considered statistically significant. For validation of the ability to predict prognosis, two independent cohorts (GSE54460 and PRAD-FR_seq_RFS) were used, and the same formula was used to calculate scores in the validation cohorts.

### Assessment of differences in clinical features and immunotherapeutic responses between PANoptosis signature score groups

The patients were grouped into high- and low-score groups, and we compared different clinical features (race, PSA level, BCR status, seminal vesicle invasion, *TP53* mutational status, and age) between them.

We collected the immunotherapeutic response data for the melanoma cohort (PMID30753825).^55^ After calculating the PANoptosis signature scores, patients were divided into high- and low-score groups to plot survival curves and visualize the relationship between the PANoptosis signature score and response to immunotherapy. Patients with immunotherapeutic responses were divided into CR, PR, SD, and PD groups. Significance was tested using Fisher’s exact test.

### Assessment of genomic alterations and HALLMARK pathways

SNV and CNV data were summarized and plotted as oncoplots. The significance of the differences in the related analysis of CNV and mutations between different PANoptosis signature score groups was assessed using the chi-squared test. GISTIC was used for CNV focal cluster visualization.^60^ After inputting the GISTIC score file and two list files of significantly amplified and deleted genes, the G scores of genomic segments could be plotted along chromosomes.

The HALLMARK enrichment score was calculated based on ssGSEA to evaluate differences in HALLMARK pathway enrichment scores between groups. Based on the PANoptosis pathway, GSEA was used to analyze the differences in the enrichment of pyroptosis, apoptosis, and necroptosis in the high- and low-score groups.

### Association between factors in the pathway mediated by ZBP1 and the PANoptosis signature score

The classic pathway for PANoptosis is mediated by ZBP1.^30^ Here, the expression levels of genes from the classic ZBP1-mediated PANoptosis pathway were compared between the high- and low-score groups using the Wilcoxon rank-sum test. Subsequently, Spearman’s analysis was used for correlation analysis between the genes and the PANoptosis signature score.

### Analysis of immune infiltrates and tumor immune escape

CIBERSORT^61^ and ssGSEA were used to quantify the relative abundance of 28 immune cell infiltrates.^62^ The ESTIMATE package was used to calculate the ImmuneScore, StromalScore, and EstimateScore. Subsequently, immune cell infiltration, ImmuneScore, StromalScore, and EstimateScore were compared between high- and low-score groups using the Wilcoxon rank-sum test.

Immune checkpoint molecules and tumor immunogenicity are involved in intrinsic immune escape.^63^ Immune checkpoints play an important role in the tumor immune system, and immune checkpoint inhibitors—one of the most promising agents in immunotherapy—are widely used in treating some tumor types.^64^^,^^65^ Twenty inhibitory immune checkpoints with therapeutic potential were selected, and the differences in their expression levels between the high- and low-score groups were assessed. In addition, differences in tumor immunogenicity, including TMB, neoantigen load, HRD, LOH, and CTA, were compared between the high- and low-score groups.

### PANoptosis signature predicts therapeutic opportunities in PRAD

Differentially expressed genes between the high- and low-PANoptosis signature score groups were calculated with DESeq2 and subjected to pathway enrichment analysis. Drug response data were downloaded from the Genomics of Drug Sensitivity in Cancer database: https://www.cancerrxgene.org/, the sample’s IC_50_ was estimated by ridge regression, and accuracy was predicted using the pRRophetic package in R. Subsequently, the correlations between the expression of PANoptosis signature genes and drug sensitivity were calculated using Spearman’s rank correlation tests. Moreover, correlations between differentially expressed genes and drug-related targets were calculated. To identify the targeted pathway or drug associated with PANoptosis, drug-target pathways were overlapped with the results of enrichment analysis of differential gene expression.

### Filtering out the PANoptosis effector cell single-cell transcriptome

Single-cell RNA sequencing profiles were obtained from two available datasets, GSE141445 and GSE157703. The complete annotation of each dataset is available at Tumor Immune Single-Cell Hub: http://tisch.comp-genomics.org.^66^ To evaluate the cellular origin of PANoptosis dysregulation, we calculated the PANoptosis signature score of each cell following a method described previously.

Single-cell transcriptome data were analyzed using the Seurat package in R, which mainly consists of the following steps: construction of object, standardization of data, clustering and dimensionality reduction, search for marker genes, etc. Finally, the cell clustering results from Seurat were annotated using the SingleR package in R. The principle of these algorithms is as follows: Spearman’s correlation coefficient between variable Gen in a single cell and each sample in the reference dataset was calculated using multiple iterations. The 80th percentile of the correlation coefficient of multiple reference samples in the same cell type was used as the annotation score for this cell type. Reference cell types annotated with a maximum score difference of 0.05 or less were retained until only two cell types remained, and the cell type with the highest correlation score was retained and annotated for this cell type.

During the use of Seurat CreateSeuratObject to build Seurat objects, the minimum number of cells was 3, and the minimum number of features was 200 for the initial filtering of genes and cells. Moreover, nFeature was (200, 6,000) and nCount was (300, 40,000) to perform data filtering again. After preliminary dimension reduction (PCA) of the data, the first 10 PCs were selected for t-SNE dimension reduction, and FindClusters was performed at a resolution of 0.5. Finally, DimPlot and FeaturePlot were used to visualize the results.

We used CreateSeuratObject of Seurat to create data objects, and thresholds were set for a minimum of 3 cells and 200 features for primary filtration of genes and cells. Then, we filtered data using nFeature = (200, 6,000) and nCount = (300, 40,000). After dimensionality reduction by PCA, the top 10 PCs were chosen for t-SNE dimension reduction, and a resolution of 0.5 was used for clustering using FindCluster. DimPlot and FeaturePlot were used for visualization.

### Quantitative real-time PCR

Total RNA was extracted using TRIzol reagent (Invitrogen) and then reverse-transcribed into cDNA using a Transcriptor First Strand cDNA Synthesis kit (Thermo Scientific, cat. no. K1622). Quantitative real-time PCR was performed using a QuantiNova SYBR Green PCR kit (QIAGEN, cat. no. 208054). The β-actin gene was used to normalize the expression of various genes. The primers used to detect mRNA levels are listed in Table S4.

### Immunohistochemical staining

Human prostate cancer tissues were obtained from West China Hospital, and immunohistochemical staining was performed to detect ZBP1 expression. Antigen was recovered with citrate buffer after dewaxing and hydration of tissue sections.

The endogenous enzyme was blocked using 3% peroxide at room temperature for 10 min. Rabbit anti-ZBP1 antibody (1:100, Affinity cat. no. DF14090) was used for incubation at 4°C overnight, and staining was developed by DAB.

The patients were divided into two groups according to high and low expression levels of ZBP1, and then clinical characteristics were compared between these two groups.

### Statistical analysis

All statistical analyses were performed using R software, v.4.1.2. We used Wilcoxon rank-sum tests for independent group comparisons. Categorical variables were compared by using the chi-squared test or Fisher’s exact test. Spearman’s analysis was used for correlation studies between quantitative variables. Survival curves were analyzed using the log rank test (generated using the KM method). Statistical significance is represented as follows: ∗p ≤ 0.05, ∗∗p ≤ 0.01, ∗∗∗p ≤ 0.001, ∗∗∗∗p ≤ 0.0001; NS, not significant.

## Data Availability

All data are freely available from public databases, and other necessary and reasonable information can be obtained from the corresponding author.

## Supplemental information

Document S1. Figures S1–S12 and Table S4

Table S1. Tumor expression profiles and clinical information

Table S2. PANoptosis pathway gene sets

Table S3. Single-nucleotide variant (SNV) data and copy-number variation (CNV) data

Table S5. Patients with tumors were separated into two subgroups by analyzing alterations in the methylation state of PANoptosis genes

Table S6. Mutation differences between the high- and low-score groups

Document S2. Article plus supplemental information
