# Supplementary material for: Construction of PANoptosis signature: Novel target discovery for prostate cancer immunotherapy
Source: Mol Ther Nucleic Acids. 2023 Jul 15;33:376–90. doi: 10.1016/j.omtn.2023.07.010 (PMC10400972; doi:10.1016/j.omtn.2023.07.010)
Supplement: Document S1. Figures S1–S12 and Table S4 [file mmc1.pdf]

OMTN, Volume *33*

Supplemental information

Construction of PANoptosis signature: Novel target discovery for prostate cancer immunotherapy

Xianyanling Yi, Jin Li, Xiaonan Zheng, Hang Xu, Dazhou Liao, Tianyi Zhang, Qiang Wei, Hong Li, Jiajie Peng, and Jianzhong Ai

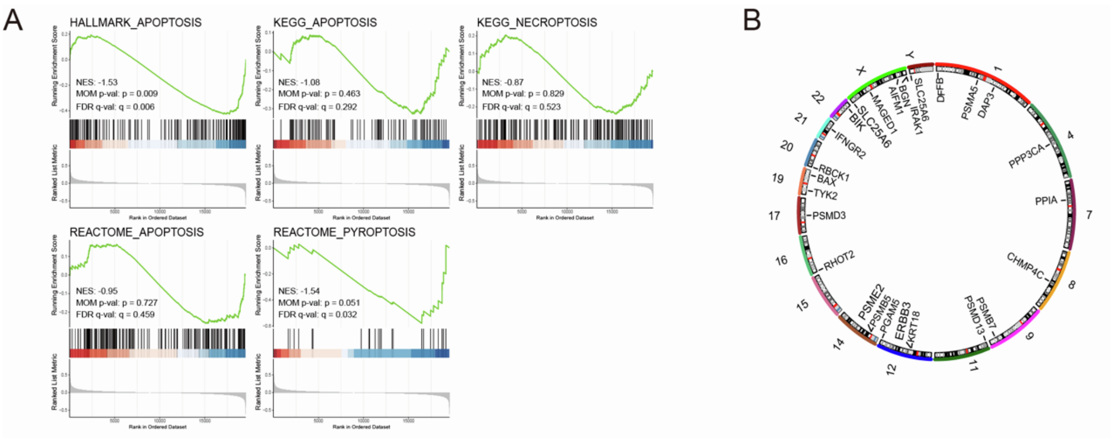

Figure S1. **Perturbations and status of gene mutation in the PANoptosis pathway in PRAD. A** The GSEA enrichment plot shows differential enrichment between age ≥60 years and age <60 years groups in "REACTOME_PYROPTOSIS, HALLMARK_APOPTOSIS, KEGG_APOPTOSIS, REACTOME_APOPTOSIS, and KEGG_NECROPTOSIS." **B** Distribution of PANoptosis pathway genes on each chromosome is presented on the Circos plot.

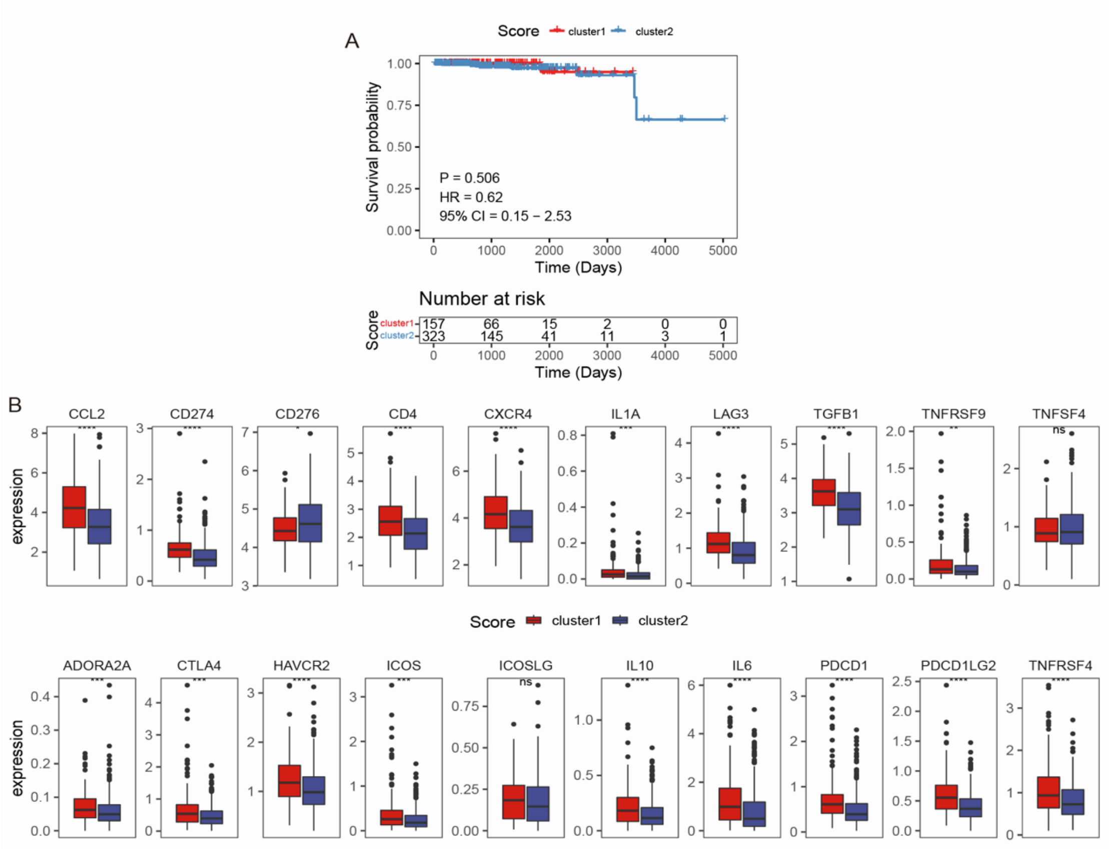

Figure S2. **Survival and expression level of immune checkpoint regulators in subgroups of patients differentiated by methylation levels. A** Survival probability between the subgroups. **B**

The expression level of immune checkpoint regulators. *$P \leq 0.05$, **$P \leq 0.01$, ***$P \leq 0.001$, ****$P \leq 0.0001$, ns: not significant.

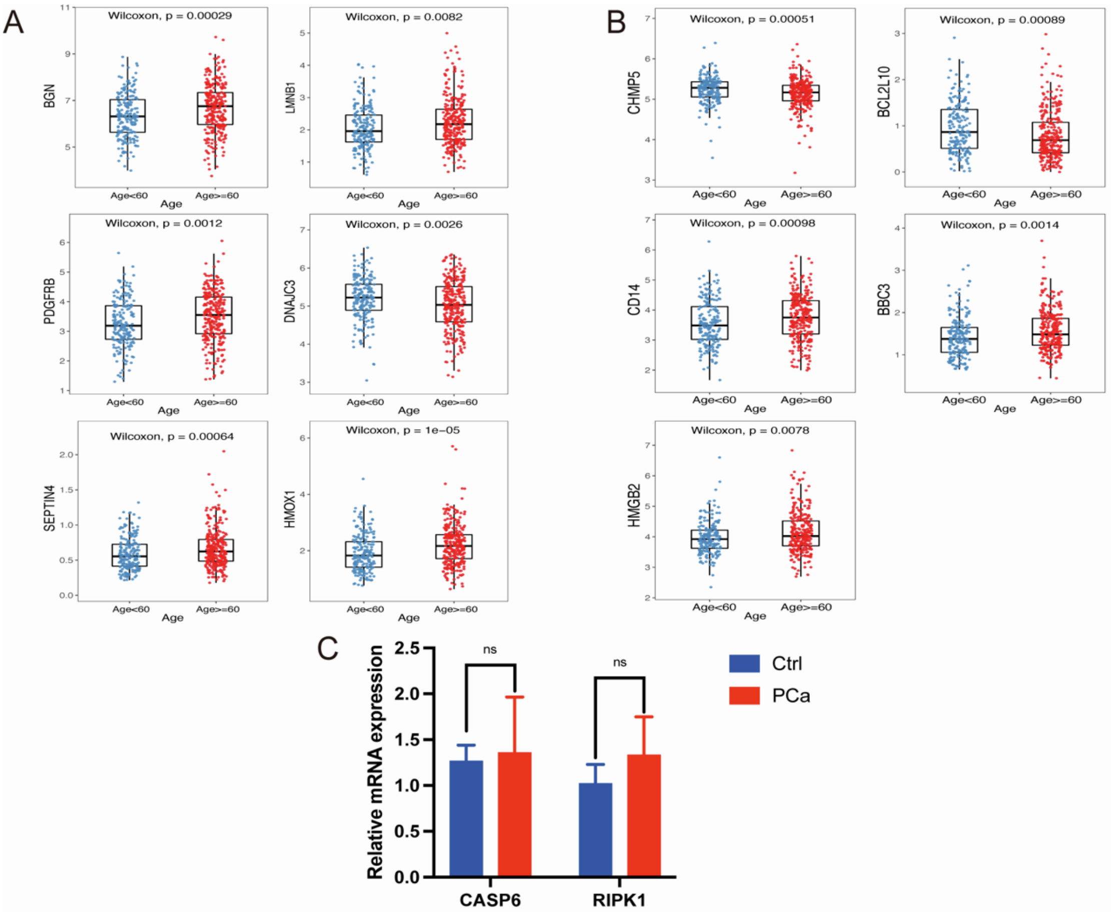

Figure S3. **Expression levels of PANoptosis pathway genes and key molecules of PANoptosis.** **A, B** Differential expression of PANoptosis pathway genes in patients aged ≥60 and <60 years. **B** qPCR for PANoptosis key molecules. ns: not significant.

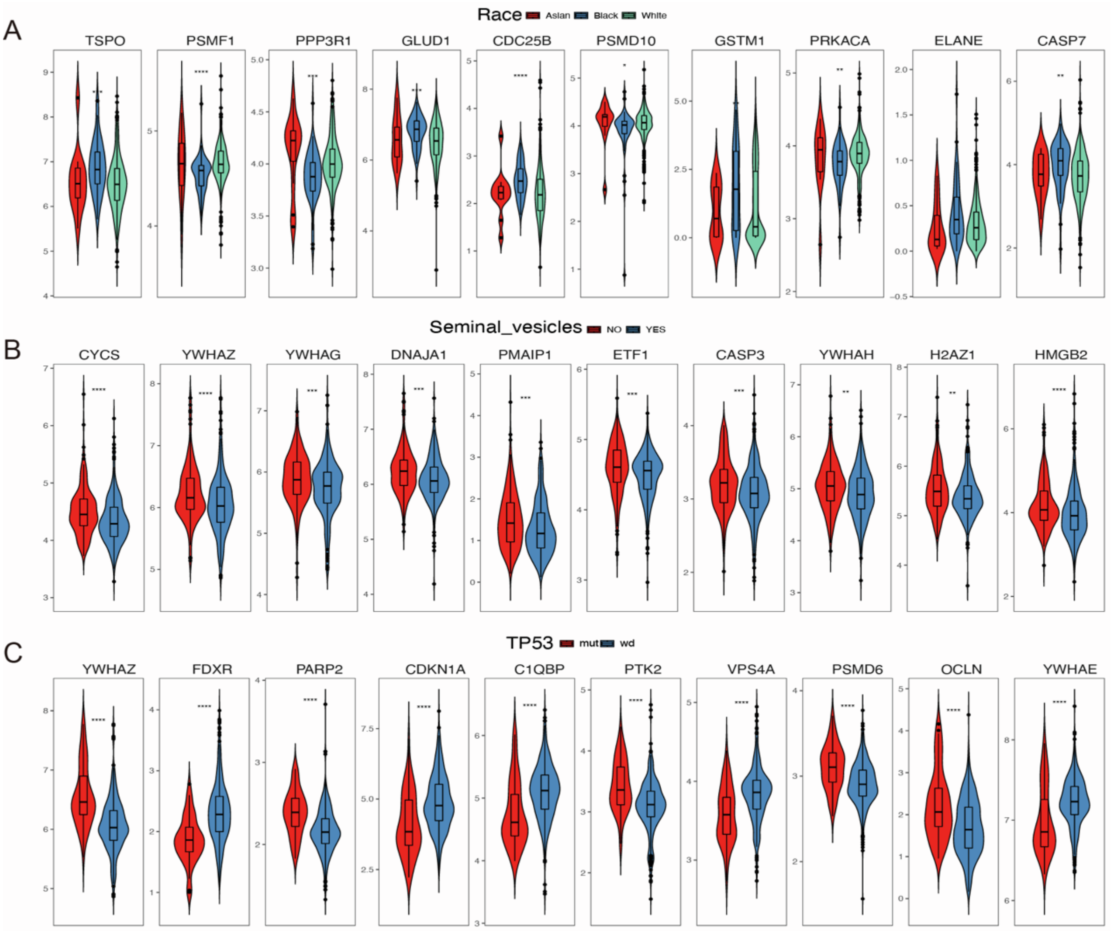

Figure S4. **Clinical Relevance of PANoptosis. A** Expression levels of PANoptosis pathway genes in various races in PRAD. **B** Expression levels of PANoptosis pathway genes in patients with and without seminal vesicle invasion. **C** Expression levels of PANoptosis pathway genes in patients with and without *TP53* mutation. $*P \leq 0.05$, $**P \leq 0.01$, $***P \leq 0.001$, $****P \leq 0.0001$.

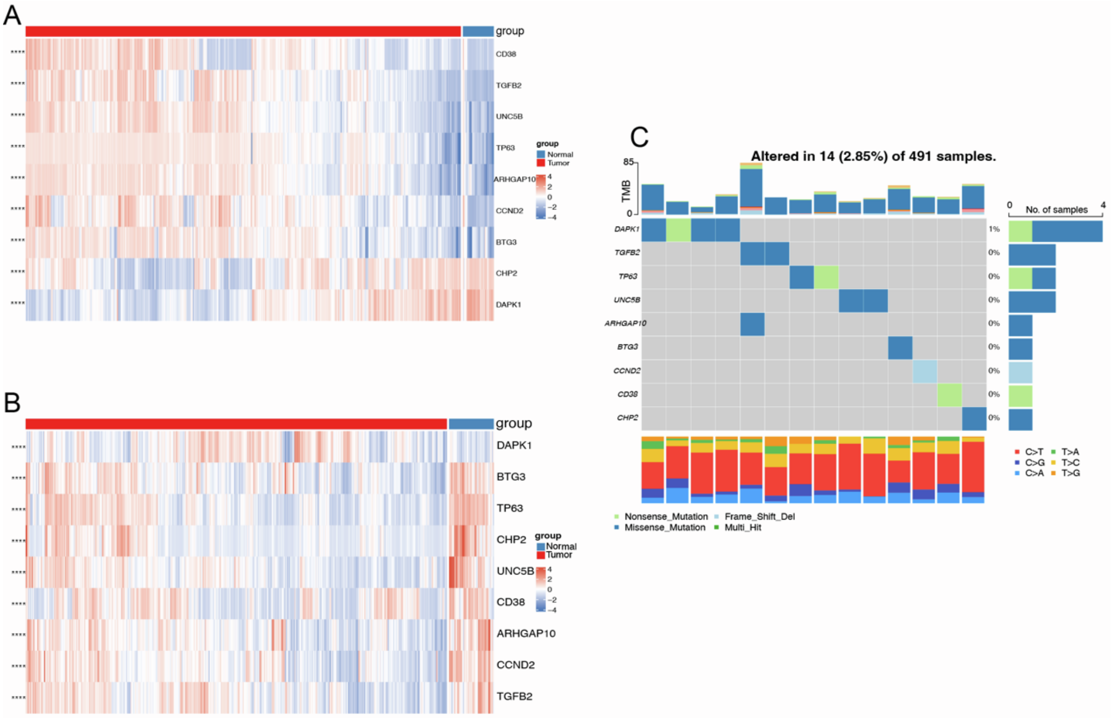

Figure S5. Overlapping of differentially methylated site genes, differentially expressed transcriptome genes, and mutated genes. A The methylation status of the nine overlapping genes. **B** Heatmap of transcriptome differential expression. **C** Mutational landscape of nine overlapping genes. ****$P \leq 0.0001$.

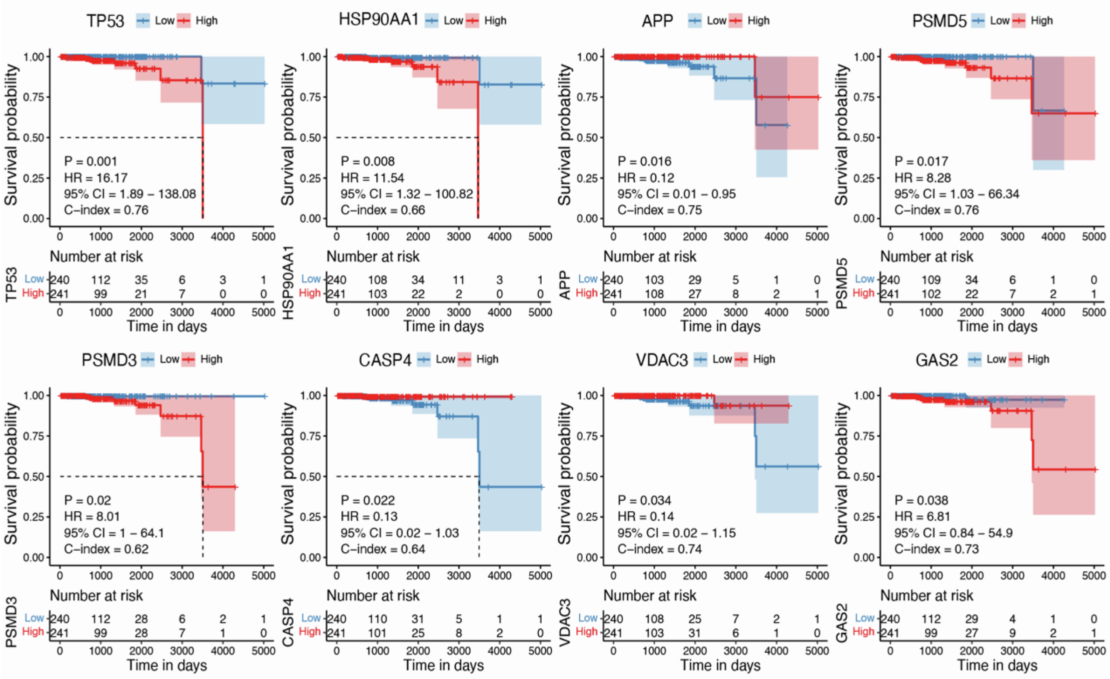

Figure S6. Expression of PANoptosis pathway genes and overall survival in patients with

PRAD. The Kaplan–Meier survival curves show eight genes whose expression is associated with overall survival.

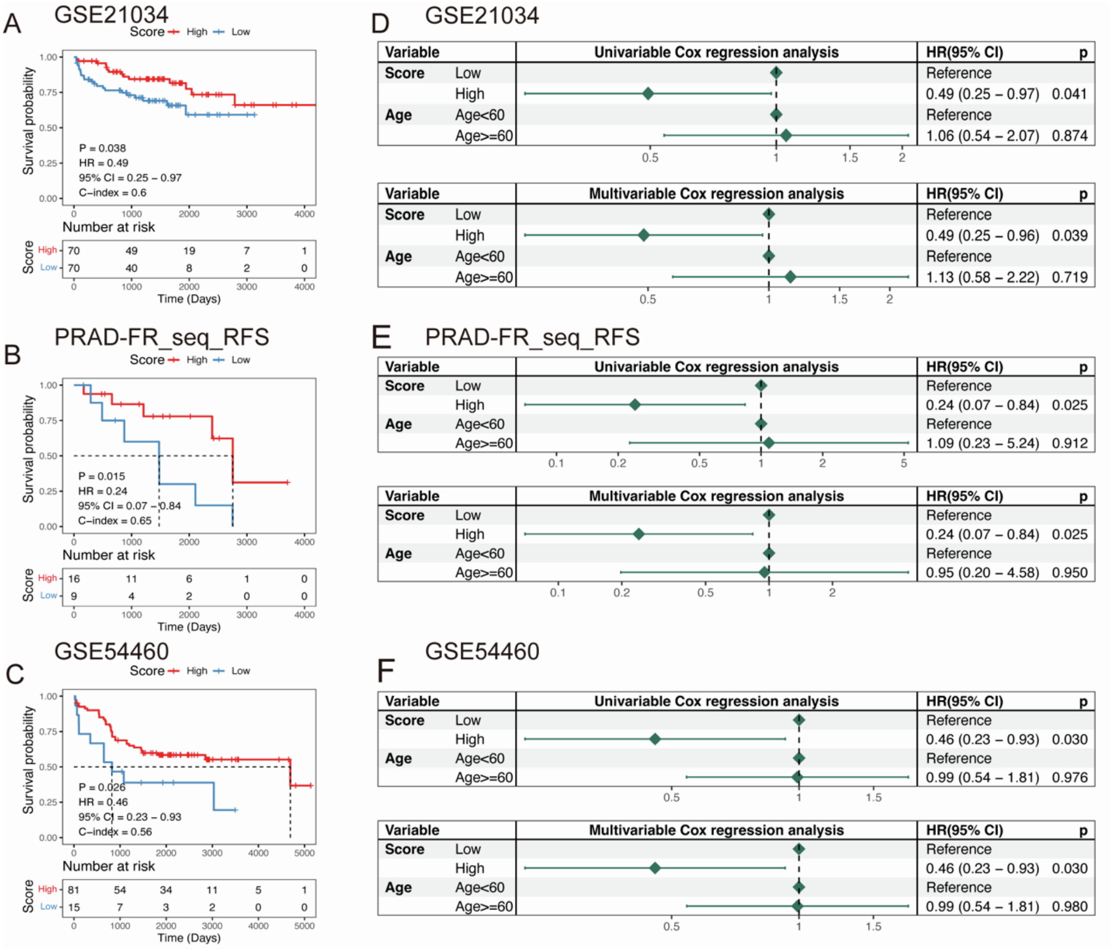

Figure S7. Validation of PANoptosis signature. A-C Kaplan–Meier survival curves show the differences in survival probability between high- and low-signature score groups in the validation cohorts (GSE21034, PRAD-FR_seq_RFS, and GSE54460). **D-F** Univariable and multivariable Cox's regression analysis for the value of prognostic prediction of PANoptosis signature score in the validation cohort.

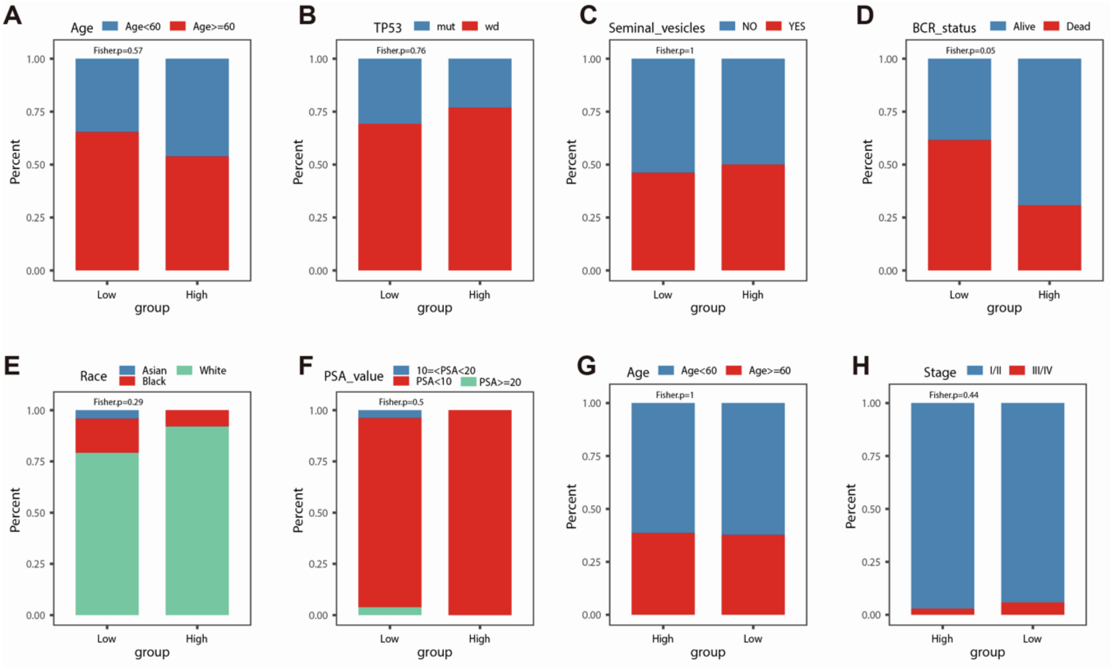

Figure S8. Clinical features in high- and low-PANoptosis signature score groups. A-F Age, TP53, seminal vesicles invasion, BCR status, race, and PSA value in high- and low-PANoptosis score groups in the TCGA cohort. **G, H** Age and clinical stage in low-PANoptosis score groups in the validation cohort (GSE21034). BCR, biochemical recurrence; PSA, prostate-specific antigen.

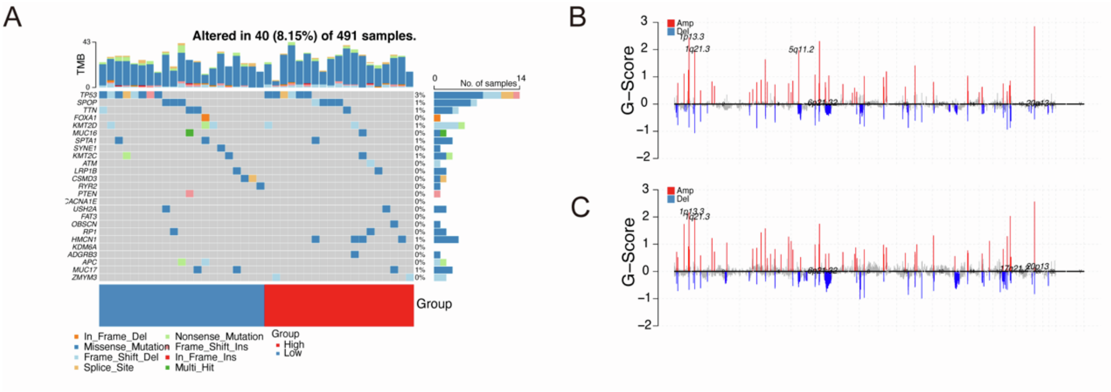

Figure S9. Genomic alterations between high- and low-PANoptosis signature score groups. A The mutational landscape of PANoptosis pathway genes altered between high- and low-risk patients with PRAD in the TCGA cohort. **B, C** G-scores of genomic segments plotted along chromosomes, red for amplifications and blue for deletions. G-scores, The Genomic Identification of Significant Targets in Cancer score.

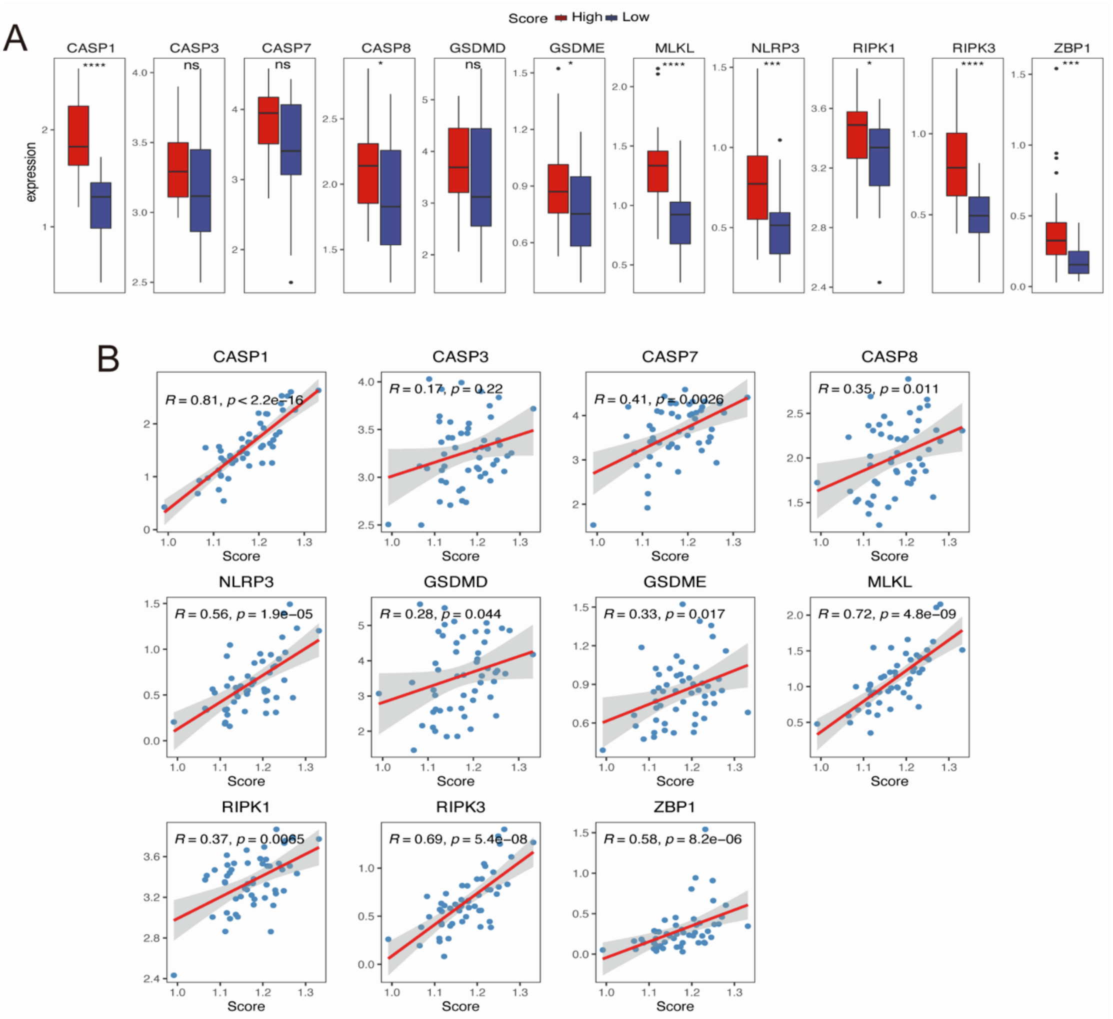

Figure S10. ZBP1-mediated PANoptosis pathway in PRAD. A Expression differences of 11 genes from the classic ZBP1-mediated PANoptosis pathway in high- and low-score groups. **B** Correlations between PANoptosis signature score and the expression level of genes involved in ZBP1-mediated PANoptosis. *$P \leq 0.05$, **$P \leq 0.01$, ***$P \leq 0.001$, ****$P \leq 0.0001$, ns: not significant.

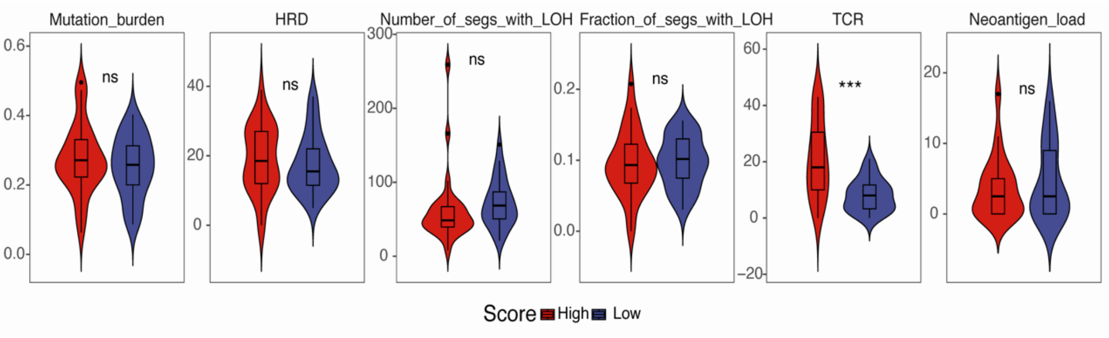

Figure S11 The role of the PANoptosis signature in tumor immune escape. TMB, HRD, LOH, CTA, and neoantigen load in the high- and low-score groups. HRD, homologous recombination deficiency; LOH, loss of heterozygosity; TCR, T cell receptor; ***$P \leq 0.001$, ns: not significant.

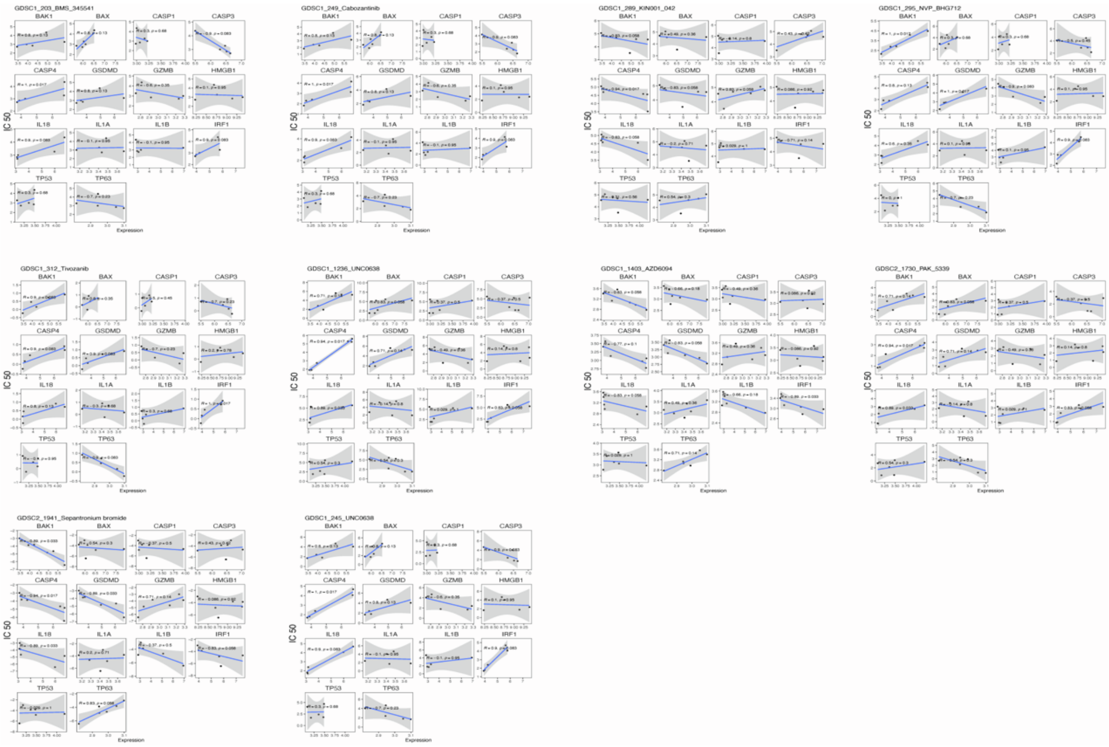

Figure S12. IC50 of drugs used in PRAD based on PANoptosis signature score. IC50, half-maximal inhibitory concentration.

Table S1 Tumor expression profiles and clinical information.

Table S2 PANoptosis pathway gene sets

Table S3 Single nucleotide variant (SNV) data and copy-number variation (CNV) data.

Table S4 Primer sequences for qPCR

Gene	Forward	Reverse
ZBP1	AACATGCAGCTACAATTCCAGA	AGTCTCGGTTCACATCTTTTGC
RIPK1	GGGAAGGTGTCTCTGTGTTTC	CCTCGTTGTGCTCAATGCAG
CASP1	GCCTTCACCATTCATGTGGAT	TTGCTCCGGGTAAAGAGACAG
CASP6	ATGGCGAAGGCAATCACATTT	GTGCTGGTTTCCCCGACAT
CASP8	TTTCTGCCTACAGGGTCATGC	GCTGCTTCTCTCTTTGCTGAA
FADD	GCTGGCTCGTCAGCTCAAA	ACTGTTGCGTTCTCCTTCTCT
β-Actin	GCGAGTACAACCTTCTTGC	TATCGTCATCCATGGCGAAC

Table S5 Patients with tumors were separated into two subgroups by analyzing alterations in the methylation state of PANoptosis genes.

Table S6 Mutation differences between the high- and low-score groups.
